# Sea surface warming and ocean-to-atmosphere feedback driven by large-scale offshore wind farms under seasonally stratified conditions

**DOI:** 10.1126/sciadv.adw7603

**Published:** 2025-11-05

**Authors:** Hyodae Seo, César Sauvage, Christoph Renkl, Julie K. Lundquist, Anthony Kirincich

**Affiliations:** ^1^ University of Hawai’i, Mānoa, Honolulu, HI, USA.; ^2^ Woods Hole Oceanographic Institution, Woods Hole, MA, USA.; ^3^ University of Bonn, Bonn, Germany.; ^4^ Johns Hopkins University, Baltimore, MD, USA.; ^5^ National Renewable Energy Laboratory, Golden, CO, USA.

## Abstract

Offshore wind farms may induce changes in the upper ocean and near-surface atmosphere through coupled ocean-atmosphere feedbacks. Yet, the role of air-sea interactions mediated by offshore wind farms remains poorly understood. Using fully coupled ocean-atmosphere-wave model simulations for seasonally stratified conditions along the US East Coast, we show that simulated cumulative reductions in wind stress due to large-scale wind farm clusters lead to sea surface warming of 0.3° to 0.4°C and a shallower mixed layer. This warming drives upward heat fluxes, destabilizing the atmospheric boundary layer and enhancing wind stress, which partially offsets wake-induced wind deficits. These wake-ocean interactions influence near-surface meteorology and air-sea fluxes, suggesting that a coupled modeling approach may be necessary for assessing potential oceanographic impacts of offshore wind developments. However, ocean coupling exerts limited influence on winds at turbine-relevant heights or within downstream wakes, resulting in minimal impact on long-term energy. These findings suggest that models without ocean coupling may be adequate for wind energy applications.

## Teaser

Offshore wind farm wakes may warm the stratified shelf ocean and modify air-sea fluxes but have minimal impact on power output.

## INTRODUCTION

**Fig. 1.**
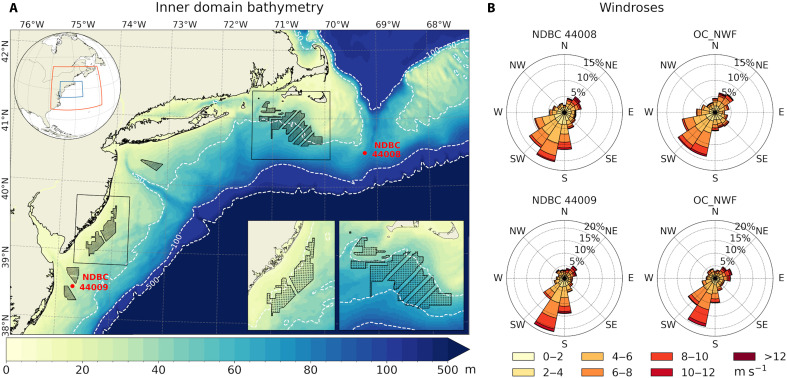
Wind energy lease areas, bathymetry, and simulated versus observed winds. (**A**) Coupled model domain with bathymetry shaded in meters. Wind energy lease areas are outlined in black, with dots representing individual turbines, totaling 1418 across all lease areas. The two largest lease areas, Massachusetts/Rhode Island (MA/RI) and New Jersey (NJ), containing 830 and 464 turbines, respectively, are enclosed in black boxes. The inset of the upper left corner shows the outer model domain (red box), which provides atmospheric boundary forcing to the coupled domain (blue box). (**B**) Wind roses illustrating wind directions (origin) and speeds (shading) from the National Data Buoy Center (NDBC) stations: Delaware Bay (Buoy 44009) and the Nantucket (Buoy 44008), compared with the unperturbed simulation (OC_NWF). NE, northeast; N, north; NW; northwest; W, west; SW, southwest; S, south; SE; southeast; E, east.

Offshore wind energy development in US coastal waters has been expanding steadily, with some commercial-scale wind farms now operational off the US East Coast (Fig. 1A). The scope of existing, planned, and proposed offshore wind projects highlights the need for fundamental research on how clusters of wind farms interact with both the meteorological and oceanographic conditions present within wind energy lease areas.

Wind turbines generate electricity by converting the kinetic energy of the wind into electrical energy. Consequently, energy extractions by turbines reduce wind speed and enhance turbulence (*1*–*3*). At both small- and large-scale deployments of offshore wind farms, these wind farm wake effects (*4*) are well documented, not only as reduced power generated within the wind farms (*5*) but also as altered near-surface meteorology and air-sea fluxes (*6*–*10*). These changes may drive oceanic and ecological responses (*11*, *12*).

For example, in the seasonally stratified North Sea and German Bight, a modeling study by Christiansen *et al.* (*13*) showed that reduced wind stress from the fixed-bottom wind farm clusters (*7*) suppresses vertical mixing in the upper ocean, leading to stronger stratification and a warming of the depth-averaged ocean temperature by ~0.1°C. These changes were shown to influence downstream ocean circulation and biogeochemical cycling (*14*). Similarly, studies along the California coast (*15*, *16*) showed that changes in wind stress profiles associated with floating wind farms may alter wind-driven upwelling circulation, with potential impacts on nutrient delivery and coastal ecosystem dynamics. On the Mid-Atlantic shelf, Miles *et al.* (*17*) reported that large wind farm clusters may affect nearshore stratification and formation of the Cold Pool (*18*), a key subsurface water mass supporting regional fisheries and ecosystems.

Despite the first-order effect of wind farm wakes on the ocean, as examined in previous studies, the role of two-way wake-ocean interaction mediated by offshore wind farms in driving oceanic and marine atmospheric boundary layer (MABL) responses remains poorly understood. Most wind wake studies rely on atmosphere-only (*19*, *20*) or atmosphere-wave coupled models (*9*, *21*, *22*), where near-surface meteorology and air-sea fluxes do not respond to sea surface temperature (SST) changes induced by wind farm wake (*6*, *7*, *10*). This contrasts with the aforementioned ocean modeling studies that highlight the dynamic nature of SST and upper-ocean variability to wake-driven wind forcing (*16*, *23*).

Similarly, marine hydrodynamic and biogeochemical models typically use prescribed wind forcing, which may limit their ability to capture two-way wake-induced feedback effects on air-sea momentum and heat fluxes—key drivers of ocean responses. While air-sea interaction has been generally recognized as important in offshore wind and oceanographic modeling (*13*, *24*, *25*), the extent to which ocean-atmosphere coupling is important and the specific conditions under which fully coupled ocean-atmosphere models are necessary remain unclear. Addressing these issues requires the use of fully coupled modeling systems that can resolve wake-induced two-way feedback.

In this study, we use high-resolution simulations with a fully coupled ocean-atmosphere-wave regional model to assess realistic scenarios involving large-scale, high-density, fixed-bottom offshore wind farms along the US East Coast. Focusing on existing wind energy lease areas (Fig. 1A), the simulations evaluate turbine-induced wake effects using the Fitch wind farm parameterization (*1*) and quantify their potential impacts on upper-ocean processes, MABL dynamics, and energy production. We define the ocean coupling effect as atmospheric responses to SST anomalies induced exclusively by wind farms. Therefore, our analysis focuses on regions near offshore wind installations. While turbine foundations can generate hydrodynamic wakes (*23*, *26*, *27*) and modify sea state and wind stress (*28*), such structural effects are not considered in this study.

We examine boreal summer, a period dominated by stable atmospheric conditions and low background turbulence (*20*, *29*, *30*), during which wake effects are pronounced (*31*, *32*). However, unstable stratification also occurs frequently in summer, allowing assessment across different stability regimes. The study region covers the seasonally stratified shelf off the coasts of Massachusetts/Rhode Island (MA/RI) and New Jersey (NJ), allowing for comparison to previous modeling studies of similar oceanographic conditions (*13*, *15*–*17*, *23*).

Simulations include fully coupled cases with and without wind wake parameterization, as well as complementary atmosphere-only simulations that exclude ocean coupling (table S1). We also assess sensitivity to the empirical parameter, α, which governs turbulence kinetic energy (TKE) generation in the Fitch scheme (Materials and Methods) (*1*). The α parameter ranges from 0 to 1.0, reflecting no to full TKE input. While the influence of α on wake characteristics and energy production is well documented (*10*, *20*, *33*, *34*), its impact on ocean responses remains not well understood. By varying α and accounting for different atmospheric stability regimes, we systematically evaluate the sensitivity of upper-ocean responses across different wake intensities.

We also quantify how wake-induced SST anomalies influence rotor layer and hub height winds, TKE, and energy production. Previous studies have shown that oceanic mesoscale SST anomalies on length scales of O (10 to 100 km) modulate air-sea heat and momentum fluxes, affecting wind shear, buoyancy, and MABL height (*35*, *36*). Hence, warmer SSTs relative to overlying air—commonly observed over warm-core eddies or the Gulf Stream—enhance upward heat flux, deepen the MABL, and accelerate near-surface winds via stronger downward momentum transport (*37*, *38*). By comparing coupled and atmosphere-only simulations, we show that SST anomalies generated by offshore wind farms exert similar effects on atmospheric stability and turbulent mixing through anomalous heat flux into the atmosphere. Our findings suggest that this feedback mechanism may be important for modeling upper-ocean and near-surface atmospheric conditions near the wind farms, but it likely has a limited influence on hub-height winds and long-term energy production.

## RESULTS

### Near-surface impacts of wind wakes

**Fig. 2.**
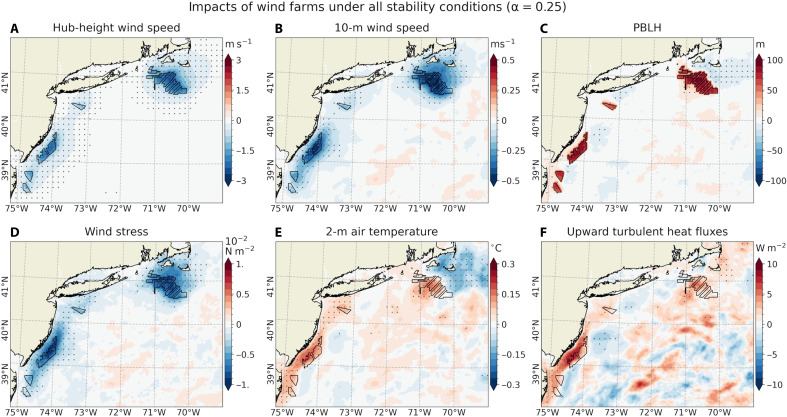
Near-surface impacts of offshore wind farms for all stability conditions with α = 0.25. Maps showing the time-averaged (JJA, 2017–2021) differences (OC_WF minus OC_NWF) for (**A**) hub height (138 m) wind speed (m s^−1^, meters per second), (**B**) 10-m wind speed (m s^−1^), (**C**) PBLH (meters), (**D**) wind stress (N m^−2^, newton per square meter), (**E**) 2-m air temperature (T2,°C)and (**F**) upward turbulent heat flux (latent + sensible; W m^−2^, watts per square meter, positive upward). Statistically significant responses at the 99% confidence level are marked with gray dots, plotted every nine grid points. All results are averaged across all stability conditions, simulated with α = 0.25.

Figure 2 illustrates the time-averaged [June-August (JJA), 2017–2021] modeled near-surface impacts of operating wind farms under all stability conditions with α = 0.25, based on coupled simulations. During summer, prevailing southwesterly winds average around 9 m s^−1^ at hub height (138 m) and 6 m s^−1^ at 10-m height (fig. S1, A and B). Within the MA/RI and NJ lease areas, wind turbines in our simulations reduce wind speeds by 2 to 3 m s^−1^ (20 to 30% of unperturbed values) at hub height and by 0.25 to 0.5 m s^−1^ (5 to 10%) at 10-m height (Fig. 2, A and B, and fig. S2, A and B). These changes are similar to those from previous studies in this region in summer (*5*, *6*, *10*, *20*) as well as over the North Sea (*7*, *8*). The North Sea studies, for example, show the wind speed reductions by about 1.8 m s^−1^ (21% reduction) at the hub height and 0.38 m s^−1^ (3 to 4%) at 10 m.

During stable conditions (fig. S3, A and B), wind speed reductions are more pronounced, and wind farm wakes extend farther downstream, particularly from the MA/RI lease areas. In contrast, under unstable conditions (fig. S4, A and B), wind speed deficits are weaker at hub height but more pronounced near the surface. This dependence on atmospheric stability is consistent with findings over the MA/RI region by Rosencrans *et al.* (*20*), reporting hub height wind deficit of 2.5 to 3.5 m s^−1^ extending downstream under stable stratification, compared to more localized deficits of 0.5 to 1 m s^−1^ confined to the wind farm region during unstable conditions. Overall, the magnitude and spatial structure of the wind speed reductions are consistent with previous findings based on Synthetic Aperture Radar observations (*32*, *39*, *40*) and numerical models (*3*, *7*, *15*, *20*).

Wind farms also influence the planetary boundary layer height (PBLH) by enhancing near-surface turbulence and modifying wind shear profiles (*10*). PBLH is defined by turbulence and buoyancy in the PBL scheme, with shear-driven turbulence dominating in stable conditions and buoyancy production of turbulence prevailing under unstable conditions (*41*). Hence, PBLH response to wind farms depends on turbine-generated turbulence (α) and the atmospheric stability conditions (*10*). In the unperturbed simulation (OC_NWF), the PBLH averages ~200 m (fig. S1C), but operating wind farms increase this by nearly 100 m (50% increase; Fig. 2C). The stronger increases in PBLH occur during stable conditions (fig. S3C), consistent with earlier studies (*10*, *42*). However, even under unstable conditions, the PBLH increases by about 50 m (fig. S4C). This increase is expected, as wind turbines serve as momentum sinks and TKE sources within the rotor-swept area (*1*).

Stability-dependent analyses with α = 0.25 (Fig. 2 and figs. S3 and S4) and sensitivity tests with α = 1.0 (figs. S5 to S7) show that increasing α leads to generally weaker near-surface wind deficits—and even slight accelerations under stable conditions (fig. S6B). This finding is consistent with earlier wake studies examining atmospheric stability effects (*10*, *22*, *34*). However, when averaged over all stability conditions, our results indicate a persistent reduction in near-surface wind speeds over the lease areas, regardless of the chosen α value. These cumulative wind speed reductions are a key driver of the associated ocean responses.

**Fig. 3.**
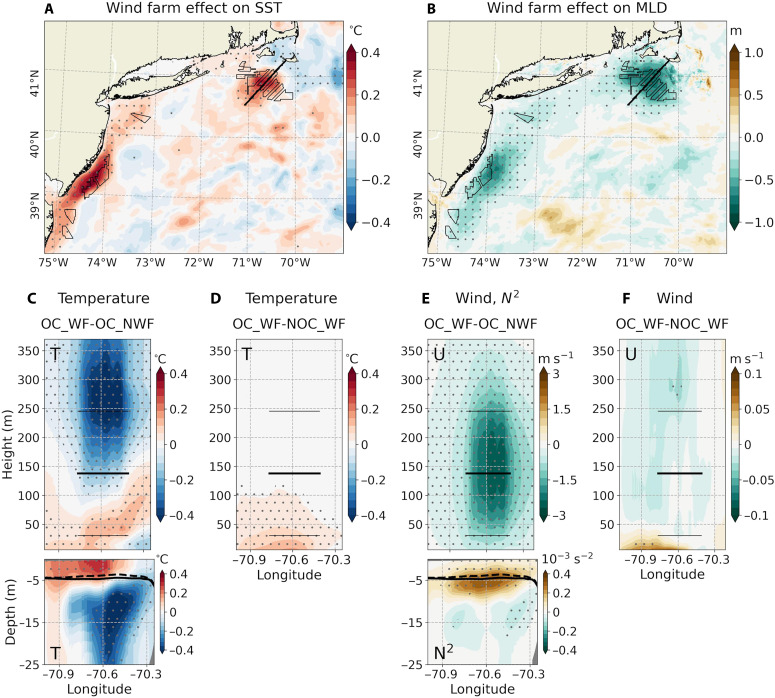
Offshore wind farm impacts on SST, mixed layer depth, and vertical ocean-atmosphere structure: MA/RI focus. (**A** and **B**) Time-averaged (JJA, 2017–2021) differences (OC_WF minus OC_NWF) for (A) SST (°C) and (B) mixed layer depth (MLD, m) under all stability conditions, simulated with α = 0.25. (**C** to **F**) Southwest-northeast cross sections across the MA/RI lease areas [black lines in (A) and (B)], showing (C) changes in atmospheric and oceanic temperatures (°C) and (E) atmospheric wind speed and oceanic Brunt-Väisälä frequency squared (*N*^2^, 10^−3^ s^−2^) due to wind farms with ocean coupling (OC_WF minus OC_NWF). Also shown are the changes in (D) atmospheric temperatures and (F) wind speed due to ocean coupling (OC_WF minus NOC_WF). The thick black lines denote the hub height (138 m), while the thin black lines denote the vertical extent of the rotor-swept area between the bottom (30.5 m) and top (245.5 m) tip of the blades. The black solid (dashed) lines denote the MLD from OC_NWF (OC_WF).

Vertical cross sections of potential temperature and wind speed provide additional insights into wake-induced impacts. With operating wind farms, time-averaged temperature above hub height drops by 0.3º to 0.4°C and wind speeds near hub height drop by 2 to 3 m s^−1^ in the MA/RI (Fig. 3, C and E) and NJ lease areas (fig. S8, C and E). Conversely, temperatures increase by 0.1° to 0.2°C, and wind speeds decrease by about 0.5 m s^−1^ near the surface. These patterns of rotor-layer cooling and near-surface warming are driven by changes in shear-driven turbulent mixing resulting from wake-modified vertical wind shear (*19*, *43*, *44*).

**Fig. 4.**
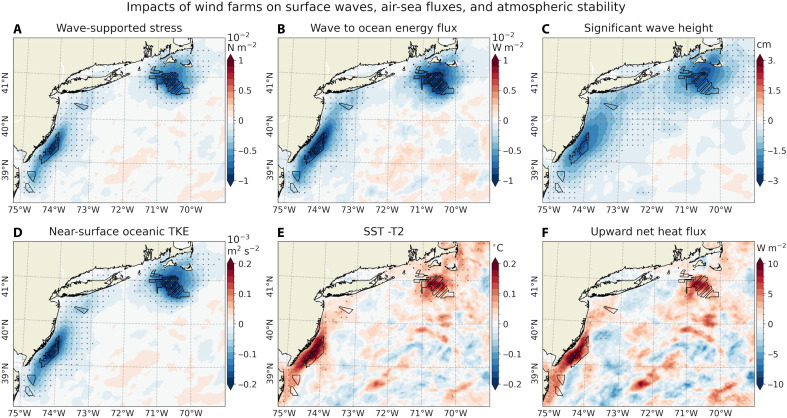
Impacts of wind farms on surface waves, air-sea fluxes, and atmospheric stability. Similar to Fig. 2, but showing (**A**) wave-supported stress (10^−2^ N m^−2^), (**B**) wave-to-ocean energy flux (10^−2^ W m^−2^), (**C**) significant wave height (centimeters), (**D**) near-surface oceanic TKE (10^−3^ m^2^ s^−2^), (**E**) SST minus T2 (°C, positive indicating unstable condition), and (**F**) upward net heat flux (watts per square meter). Results are averaged across all atmospheric stability conditions and simulated with α = 0.25.

The time-averaged near-surface wind speed deficits—averaged across different stability regimes—lead to a reduction in wind stress within the wind farm regions, with decreases of 10 to 20% in the lease areas and 5 to 10% downstream (Fig. 2D and fig. S2D). Wind stress reduction patterns and their stability dependence mirror those of the 10-m wind field. Similar reductions are found in sea-state and surface wave–related variables derived from the wave component of the coupled model (Materials and Methods). These include wave-supported stress, representing momentum flux from wind to surface waves; wave-to-ocean energy flux, which contributes to wave-induced mixing in the surface ocean; and significant wave height, a measure of wave energy (Fig. 4, A to C). The reduction in wind stress results in decreased surface wave energy and weaker wave-induced turbulent mixing (*21*, *22*, *45*, *46*), thereby reducing near-surface ocean TKE (Fig. 4D), consistent with the findings of Christiansen *et al.* (*13*).

Figure 3 (C and E) and fig. S8 (C and E) show vertical profiles of ocean potential temperature and the Brunt-Väisälä frequency squared (*N*^2^), which indicates ocean density stratification. During summer, the observed and modeled Mid-Atlantic Bight is strongly stratified (*17*, *47*), with the mixed layer depth (MLD), estimated from the observational data using a 0.03 kg m^−3^ density threshold, remaining less than 5 m near the wind farms (*48*). With the wind farms in place, the MLD decreases by about 1 m, a 20% reduction from the unperturbed conditions (Fig. 3B). Ocean warming is concentrated within the mixed layer, while cooling occurs below, indicating enhanced upper-ocean stratification. The greatest stratification increase is found at the base of the mixed layer. These patterns of near-surface ocean warming and increased stratification are consistent with reductions in wind stress, TKE, and turbulent mixing (*13*, *23*, *49*). The resulting changes in upper-ocean processes lead to SST warming of 0.3° to 0.4°C, most pronounced near the large wind farms, including those in the MA/RI and NJ lease areas (Fig. 3A). In the following sections, we examine the characteristics of these SST warming patterns and their feedback effects on the atmosphere in greater detail.

**Fig. 5.**
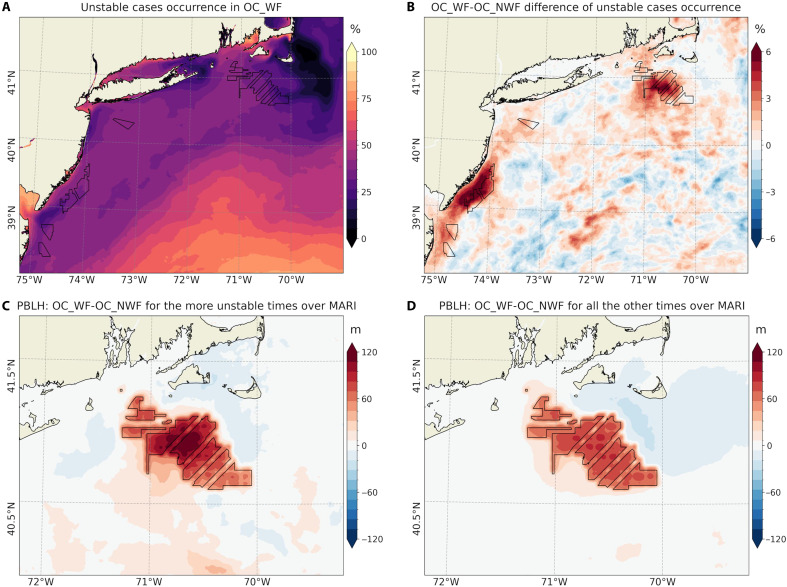
Changes in atmospheric stability due to wake-induced ocean warming and its impacts on PBLH. (**A**) Percentage occurrence (%) of unstable conditions during JJA, 2017–2021 in the unperturbed simulation (OC_NWF). (**B**) Change in the percentage occurrence of unstable conditions due to wind farms. (**C**) Composite averaged change in PBLH (m) during the 5% of the time when atmospheric stability transitions to unstable over the MA/RI lease areas, as identified in (B). (**D**) As in (C) but for the remaining 95% of the time when stability does not change.

Wind farms also affect upward turbulent (latent + sensible) heat fluxes (*7*, *8*) and the atmospheric boundary layer stability. Our simulations show that the warm SST response is associated with the weakly positive anomalies of 3 to 5 W m^−2^ off MA/RI and up to 10 W m^−2^ off NJ (Fig. 2F; see also net heat flux in Fig. 4F). Here, positive fluxes indicate atmospheric heating and ocean cooling. Note that the SST warming exceeds 2-m air temperature (T2) by up to 0.2°C (Fig. 4E). Hence, this upward heat flux is associated with a more unstable MABL, as evident in the changes to the vertical atmospheric temperature profile (Fig. 3C and fig. S8C). We find that SST warming is associated with a 5% increase in unstable atmospheric conditions near the lease areas (Fig. 5). If compositing only the 5% of the times when the MABL is more unstable in OC_WF than OC_NWF, PBLH increases by more than 120 m—compared to a 50-m increase in the remaining 95% of cases (Fig. 5, C and D). The increase in PBLH under unstable conditions is likely due to buoyancy-driven TKE production, as supported by the elevated TKE over regions of SST warming (fig. S9).

In models without ocean coupling, changes in turbulent heat flux are primarily attributed to wake-induced modifications in near-surface meteorological conditions. In such cases, air-sea heat fluxes are often directed into the ocean, associated with near-surface air temperature increases and the resulting stable atmospheric boundary layer (*6*, *7*, *10*). In contrast, our coupled model results indicate that SST warming can exceed 2-m air temperature warming, leading to upward (ocean-to-atmosphere) heat fluxes and a tendency toward a more unstable atmospheric boundary layer.

### SST warming response to wind wakes

**Fig. 6.**
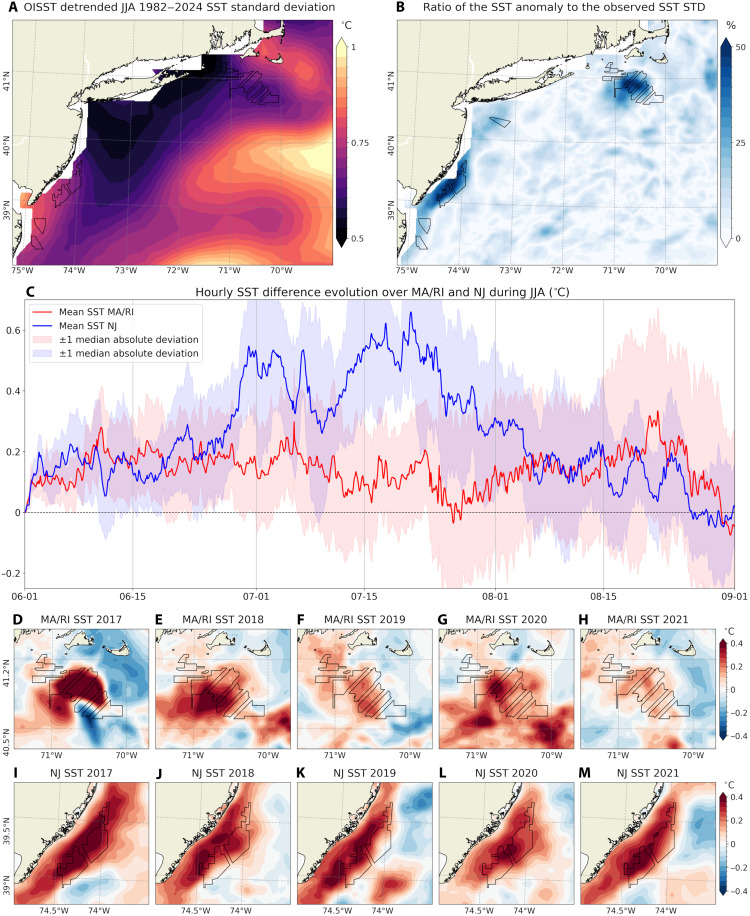
Interannual SST variability, wake-driven SST anomalies, and spatiotemporal evolution. (**A**) Spatial distribution of the standard deviation of detrended summertime (JJA, 1982–2024) SST anomalies (°C), derived from the 0.25° NOAA Optimum Interpolation (OI) SST V2 dataset (*86*). (**B**) Spatial map of the percentage ratio (%) of the modeled SST response induced by wind farms relative to the interannual variability shown in (A). (**C**) Hourly time series of the five-summer averaged SST anomaly over the MA/RI (red) and NJ (blue) lease areas, with shading indicating ±1 median absolute deviation across the five-summer period. (**D** to **H**) JJA-averaged SST anomalies over the MA/RI lease areas for each simulation year, showing interannual variability. (**I** to **M**) As in (D) to (H) but for the NJ coast.

In the coupled model, SST increases near offshore wind farm regions (Fig. 3A) are associated with wake-induced reductions in wind stress and near-surface turbulent mixing in the ocean. Although the magnitude of this warming (0.3° to 0.4°C) represents a small fraction of the large annual SST cycle in the Mid-Atlantic Bight (7° to 25°C) (*50*), it accounts for ~50 to 60% of the detrended summertime interannual SST variability observed on the US East Coast outer continental shelf (Fig. 6, A and B). This section provides further characterization of the SST responses.

Figure 6C shows the hourly time series of the SST differences (OC_WF minus OC_NWF) over the MA/RI and NJ lease areas. The figure highlights a rapid spin-up of SST warming in response to changes in wind stress. Following this initial adjustment, the SST warming signal exhibits pronounced day-to-day, submonthly, and subseasonal variability. Considerable spatial and interannual variability is also found in the seasonally averaged (JJA-mean) SST response. Figure 6 (D to M) displays maps of year-to-year variability in the JJA-mean SST responses. In MA/RI, the summers of 2017, 2018, and 2020 show stronger SST warming, reaching up to 1°C, while 2019 and 2021 show modest or muted warming (about 0.1°C). In the NJ region, warming extends broadly along the coast, with stronger warming in 2017, 2019, and 2021 and weaker warming in 2018 and 2020. These differences in interannual variability between MA/RI and NJ suggest region-specific mechanisms driving the SST responses. Similar patterns of widespread SST warming have been reported in simulations along the California coast in association with floating offshore wind farms (*16*).

**Fig. 7.**
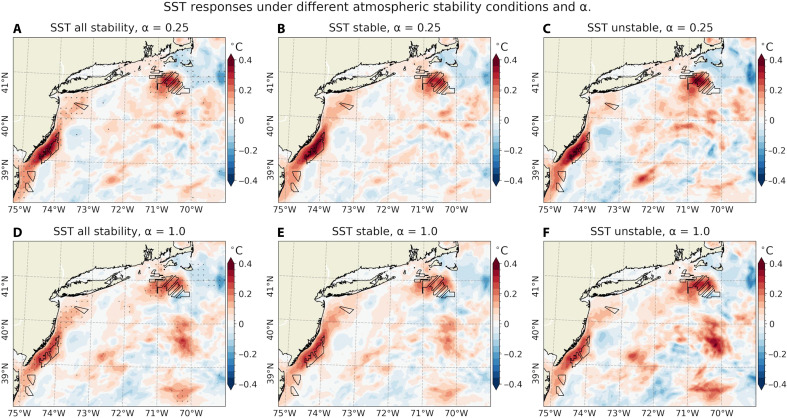
SST responses under different atmospheric stability conditions and TKE factors. Time-averaged (JJA, 2017–2021) SST (°C) response (OC_WF minus OC_NWF) for (top) α = 0.25 and (bottom) α = 1.0, under (**A** and **D**) all stability conditions, (**B** and **E**) stable, and (**C** and **F**) unstable conditions only.

Despite the spatio-temporal variability and variations in magnitude, the time-averaged SST responses are consistent across all combinations of atmospheric stability regimes and α values considered in this study (Fig. 7). The SST warming appears consistently in all cases and is spatially well aligned with the largest offshore wind farm areas, including those in MA/RI and NJ. This consistent SST response corroborates time-averaged reductions in near-surface wind speed and stress (Fig. 2, B and D). While wind wake characteristics are known to be sensitive to prevailing atmospheric stability and chosen TKE factor, SST warming shows much weaker sensitivity to these factors.

Changes in SST are also simulated beyond the continental shelf and in the deep ocean. However, these far-field responses are generally smaller in magnitude and spatially less coherent than those occurring adjacent to large wind farms. These regions exhibit far greater oceanic internal variability than the shelf ocean (Fig. 6A). Robust attribution of the forced responses in remote regions with enhanced internal variability will likely require longer simulations. Instead, our analysis focuses on coastal areas adjacent to wind farms, where the ocean response is consistent, and the underlying physics that determines upper-ocean processes, air-sea fluxes, and MABL properties is better understood in the literature.

### Ocean mixed layer temperature balance

The increase in SST, despite enhanced upward surface heat flux, indicates that upper-ocean processes may contribute to sustaining the surface warming. To explore the physical mechanisms involved, this section presents a detailed analysis of the online ocean mixed layer temperature (MLT) budget calculations (Materials and Methods).

**Fig. 8.**
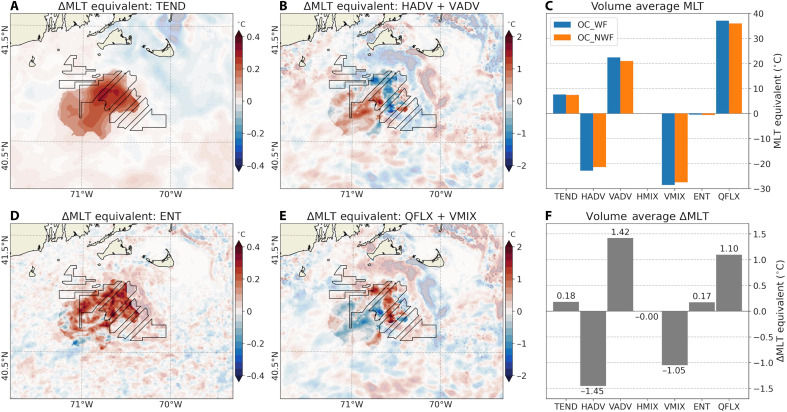
Changes in mixed layer temperature equivalents in MA/RI. Maps showing difference in time-integrated (JJA, 2017–2021) MLT equivalents (°C) between OC_WF and OC_NWF attributed to: (**A**) total tendency (TEND), (**B**) horizontal advection (HADV) plus vertical advection (VADV), (**D**) entrainment (ENT), and (**E**) surface heat flux (QFLX) plus vertical mixing. (**C**) Area-averaged MLT equivalents in OC_WF (blue) and OC_NWF (orange) and (**F**) their differences (OC_WF minus OC_NWF). Regions outside the control volume used for the budget calculation are masked. Note the different color scales in (A), (B), (D), and (E).

In the unperturbed simulations (fig. S10), the warming of the MLT across the shelf is primarily driven by surface heat flux (QFLX) warming (*51*). This warming is partially offset by cooling from VMIX, with a secondary contribution from entrainment (ENT) cooling. Horizontal heat flux divergence (HADV) cools the MLT (Fig. 8C), which is largely offset by the warming due to vertical temperature advection at the base of the mixed layer (VADV).

Given the differences in physical mechanisms driving the SST responses, we analyze the MLT budget separately for the MA/RI and NJ regions. Each of the budget terms in Eq. 3 is time-integrated to obtain MLT equivalents (in units of °C) and spatially averaged over the corresponding control volumes (Materials and Methods).

In the MA/RI region, the MLT increases by ~0.18°C, primarily due to reduced ENT cooling (0.17°C) (Fig. 8F). ENT cooling is associated with the deepening of the mixed layer, entraining colder subsurface water into the mixed layer. Therefore, reduced ENT cooling under wind farms is consistent with a shallower mixed layer (Fig. 3B). Additional warming is driven by VADV (1.42°C), which is almost entirely balanced by HADV cooling (−1.45°C) (Fig. 8F). QFLX contributes to net warming (1.10°C), and this is despite the fact that the net heat flux change is upward (i.e., ocean cooling; Fig. 4F). This is because QFLX depends inversely on MLD in addition to net heat flux, $Q_{\text{net}}$ (Eq. 3), and changes in MLD (20%) are relatively larger than changes in $Q_{\text{net}}$ . The warming from QFLX is, however, nearly offset by cooling due to enhanced VMIX (−1.05°C). Hence, when averaged in time and control volume, these two contributions nearly cancel each other out.

In the NJ region, the maximum MLT warming occurs slightly shoreward of the wind farms (fig. S11A), where MLD changes are minimal (Fig. 3B). Hence, an increase in upward surface heat flux (Fig. 4F) results in a net QFLX-induced cooling of −0.56°C. Meanwhile, reduced wind stress weakens VMIX, diminishing VMIX-driven cooling by 1.55°C. Combined, these two effects yield a net warming of the MLT by 0.99°C. Within the NJ lease areas, the MLD shoals by ~20%, which weakens ENT cooling and contributes an additional 0.1°C of MLT warming. VAVD through the base of the mixed layer adds warming (4.69°C), indicating reduced upwelling, while HADV induces cooling (−5.55°C). The combined effect of total advection (HADV + VADV) is a net cooling of −0.86°C. With only minor changes in horizontal mixing (HMIX) (−0.01°C), the imbalance results in a net MLT warming of 0.21°C.

Figure S12 illustrates the potential changes in upwelling conditions along the NJ coast. It shows that upwelling-favorable alongshore wind stress is weakened shoreward of the wind farms (fig. S12A), compared to the unperturbed case. The cross-shore section shows that, in the absence of wind farms, the 21.6°C isotherm outcrops 20 to 30 km offshore, while with the wind farms, it remains at the subsurface, indicating near-surface warming (fig. S12B). This pattern is consistent with a reduction in cross-shore Ekman transport, the most pronounced shoreward of the NJ lease areas (fig. S12C).

A previous study (*13*) attributed ocean warming over seasonally stratified North Sea shelf to reduced shear-driven mixing caused by wind farms. Our findings are consistent with this mechanism in the NJ region, where VMIX is the dominant factor in MLT warming. In the MA/RI region, on the other hand, the primary driver is reduced ENT cooling associated with a shallower mixed layer. Previous coastal upwelling studies (*16*, *17*) have also demonstrated potential changes in upwelling circulation and cross-shelf circulation due to altered wind stress. Our results support these findings, showing that both VADV and HADV play key roles in MLT warming.

### Ocean-to-atmosphere feedback

As the SST responses emerge following wind stress reductions and changes in the ocean mixed-layer processes, they can also continually influence the MABL by transferring anomalous heat from the ocean to the atmosphere. To quantify this two-way interaction resulting from wake-induced ocean coupling, we compare the OC_WF simulation (ocean coupling with wind farm) with a complementary atmosphere-only simulation, NOC_WF (no ocean coupling with wind farm), in which the significant warm SST anomalies in the lease areas are selectively removed from the lower boundary SST forcing in WRF (Materials and Methods). This experimental setup allows for a direct attribution of atmospheric differences between the two simulations to the SST response induced by wind wakes.

**Fig. 9.**
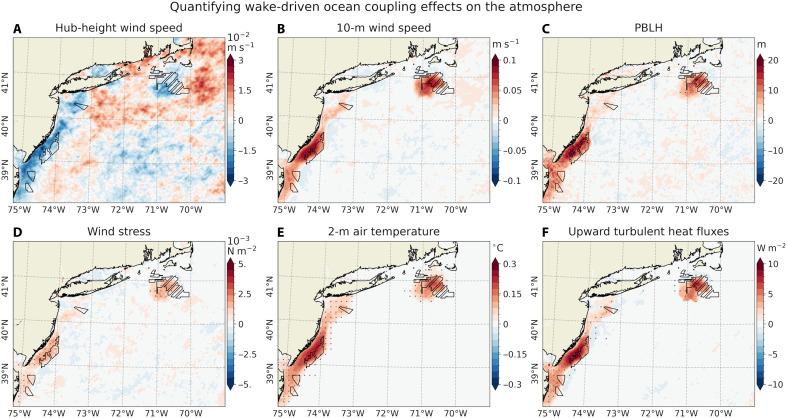
Quantifying wake-driven ocean coupling effects on the atmosphere. (**A** to **F**) Similar to Fig. 2 but showing the differences (OC_WF minus NOC_WF) under all stability conditions, simulated with α = 0.25. Note that the color scale in (A) differs from that of Fig. 2A, with a range of ±0.03 m s^−1^ compared to ±3 m s^−1^.

In response to warm SSTs, the PBLH in OC_WF increases by ~20 m relative to NOC_WF (Fig. 9C). This increase corresponds to roughly 10% of the climatological PBLH (200 m; fig. S1C), and about 20% of the total PBLH increase attributed to wake effects (100 m; Fig. 2C). Associated increases in TKE over the warm SSTs are also obtained throughout the rotor layer, with maximum enhancements between the hub height and near the top blade tip (fig. S9E).

Similarly, both 10-m wind speed and surface wind stress (Fig. 9, B and D) increase over warm SST regions—by ~10 and 20%, respectively—relative to the wake-induced reductions seen in Fig. 2 (B and D). These enhancements corroborate the mesoscale air-sea interaction mechanism (*35*, *52*), in which warmer SSTs increase downward momentum transport, accelerating near-surface wind speeds while reducing wind aloft (*37*, *38*). However, at hub height and across the rotor-swept layer, changes in wind speeds are small—about 0.01 m s^−1^ or less than 1% of the wake-induced hub height wind speed deficit (Figs. 3E and 9A and fig. S8E). Thus, SST-induced wind speed modifications are confined mainly to the atmospheric surface layer, with minimal impact at turbine-relevant heights.

The upward turbulent heat flux attributed to ocean coupling ranges from 5 to 10 W m^−2^ (Fig. 9F), comparable to the total heat flux change seen from the fully coupled case (Fig. 2F). This suggests that most of the upward heat flux in the coupled simulation may stem from SST warming. Similarly, near-surface air temperature increases by 0.2°C, consistent with the fully coupled case (Fig. 2E), further supporting the SST-driven nature of the near-surface atmospheric warming. Notably, warming above the bottom blade tip (30.5 m) seen in the coupled run is absent in the uncoupled simulation (Fig. 3, C and D, and fig. S8, C and D), suggesting that it is not induced by SST warming. Similar warming near the bottom blade tip has also been reported in previous atmosphere-only studies (*6*–*8*).

Hence, the most notable differences in wind speed and air temperature due to ocean coupling are found within the atmospheric surface layer, extending up to ~50 m (Fig. 3, D and F, and fig. S8, D and F). These surface-intensified changes suggest that the influence of SST feedback is relatively shallow. Above this layer, particularly within the rotor region, atmospheric changes appear to be more strongly influenced by wake-induced variations in shear and turbulence. In addition, the downstream reductions in PBLH, wind speed, wind stress, and surface air temperature seen from the coupled simulations (Fig. 2) are largely absent, suggesting that, on average, the effects of ocean coupling are spatially limited to wind farm regions, with no strong downstream impact.

The slight reduction in wind speed at turbine-relevant heights implies a small impact of ocean coupling on long-term wind energy production over the study period (JJA, 2017–2021). The total accumulated energy generation in the coupled simulation is only 0.13% lower than in the uncoupled run for the MA/RI region and 0.24% lower in the NJ region (table S3). While these differences are not entirely negligible, they indicate that, under the conditions analyzed, ocean-atmosphere coupling exerts a limited influence on long-term power generation and is unlikely to substantially affect wind energy resource assessments.

The weak SST-induced effects on winds at turbine-relevant heights in this study should not be interpreted as evidence that SST fields are unimportant in wind energy modeling. Previous research has shown that hub-height wind speeds, wind resource assessments, and power outputs can be sensitive to background SST distributions (*53*, *54*, *55*) likely through mechanisms different from those examined here. A key distinction in this study is the specific definition of “ocean coupling,” which refers solely to the feedback effect of local SST anomalies generated by wake-induced reductions in wind stress. While this targeted definition enables a focused evaluation of wake-driven local air-sea feedback, broader-scale or longer-term ocean-atmosphere interactions may also influence energy production and warrant further investigation.

## DISCUSSION

**Fig. 10.**
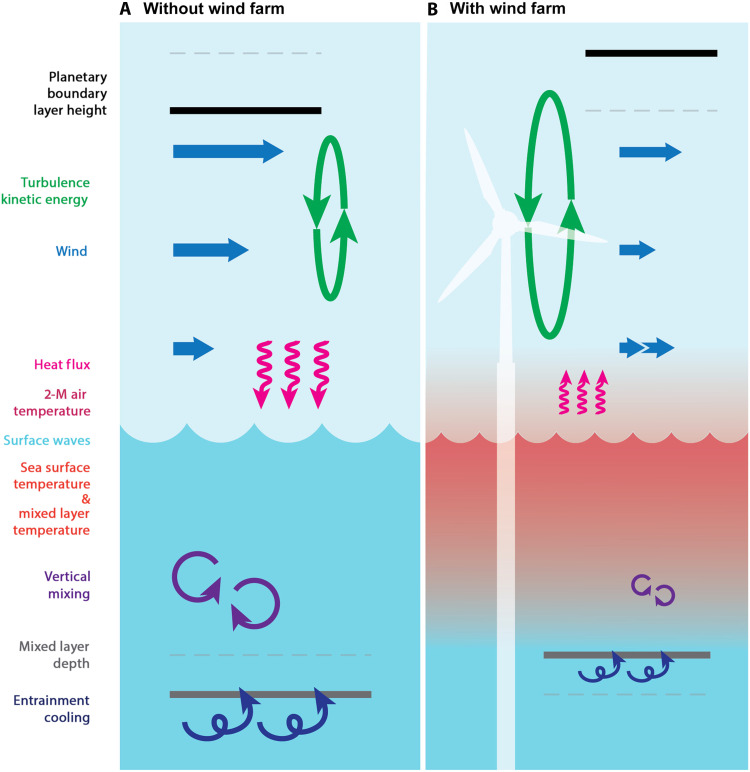
Schematic summary of potential upper-ocean and lower-atmosphere interaction processes near offshore wind farms. (**A**) Without wind farms, stronger winds aloft and weaker near-surface winds generate vertical wind shear and atmospheric turbulent kinetic energy (TKE). In the ocean, downward surface heat flux warms the ocean, and vertical mixing redistributes heat by mixing warm near-surface waters with cooler subsurface waters entrained from below the mixed layer. (**B**) With wind farms in place, wake-induced turbulence enhances atmospheric TKE and raises the planetary boundary layer height (PBLH), while wind speeds at both hub height and near the surface are reduced. Weakened wind stress suppresses surface wave activity and wave-driven mixing, resulting in a shallower mixed layer and reduced entrainment cooling. This leads to warming of both the mixed layer temperature and the sea surface temperature. As the sea surface warming exceeds the near-surface air temperature warming, the surface heat flux becomes weakly and anomalously upward into the atmosphere. The resulting unstable stratification further enhances atmospheric TKE and PBLH. These destabilized atmospheric boundary layer conditions promote downward momentum transport, accelerating near-surface winds and partially offsetting wake-induced wind speed reductions.

This study examines potential sea surface warming responses induced by cumulative reductions in wind stress due to wind farm wakes in the seasonally stratified Mid-Atlantic Bight shelf region (Fig. 3A). Figure 10 schematically summarizes the key processes contributing to the upper-ocean response and the resulting atmospheric feedback, comparing near-surface ocean-atmosphere interactions under unperturbed conditions (Fig. 10A) with those potentially mediated by operating wind farms (Fig. 10B). On average, wind farm wakes reduce near-surface wind speed and stress (Fig. 2), thereby lowering upper-ocean TKE (Fig. 4). These changes lead to a shallower mixed layer (Fig. 3B), enhanced stratification (Fig. 3E), and altered upwelling (fig. S12). SST warming exceeds the increase in near-surface air temperature (Fig. 4E), resulting in anomalously upward heat flux and a tendency toward more unstable atmospheric conditions. In our simulations, ocean coupling increases the frequency of unstable conditions by ~5% over the wind farm areas, contributing to a 20% increase in PBLH compared to the case without ocean coupling (Figs. 5 and 9).

SST warming emerges within days of simulation onset (Fig. 6C), consistent with earlier findings (*13*). Once established, the SST anomaly patterns show substantial temporal variability. This variability differs between the MA/RI region (Fig. 6, D to H) and the NJ coast (Fig. 6, I to M), suggesting that distinct physical mechanisms govern the SST warming responses. The analysis of online ocean MLT budget calculations reveals that, in MA/RI, MLT warming is primarily driven by reduced entrainment cooling associated with a shallower MLD (Fig. 8). In NJ, reduced vertical mixing and weakened upwelling, linked to weaker alongshore wind stress, are identified as the dominant contributors to MLT warming (figs. S11 and S12). The SST warming of 0.3° to 0.4°C is small compared to the regions’ large annual cycle (7° to 25°C), but it accounts for ~50 to 60% of the interannual variability (Fig. 6, A and B). Moreover, the time-averaged SST responses are consistent across varying atmospheric stability regimes and values of the empirical turbulence parameter in the wind farm parameterization (Fig. 7).

A comparison between fully coupled and atmosphere-only simulations—excluding ocean feedbacks—reveals that SST warming enhances upward heat fluxes and wind stress (Figs. 9 and 10B), consistent with the mesoscale air-sea interaction mechanism observed over warm SST anomalies. Since surface fluxes play an important role in driving both physical and biogeochemical ocean processes, these results suggest that including coupled ocean-atmosphere interactions may be necessary for assessing hydrodynamic and ecological responses to offshore wind development.

In our simulations, the most noticeable effects of ocean coupling are confined to the atmospheric surface layer, below turbine-relevant heights. In contrast, changes in wind speed across the rotor layer and at hub height are minimal, with reductions of less than 1% (Figs. 3F and 9A). This slight decrease in hub height winds leads to a correspondingly small reduction in long-term energy production (0.13 to 0.24%) (table S3). Moreover, the influence of ocean coupling appears to be spatially limited to the wind farm regions, with no discernible downstream influence. These simulation results suggest that, under the examined conditions, atmosphere-only or atmosphere-wave coupled models without ocean coupling may be adequate for estimating wind resources, power output, and wind farm wake effects.

The SST warming is reminiscent of land surface temperature (LST) warming induced by onshore wind farms in stably stratified conditions (*1*, *43*, *56*–*60*). Previous studies report robust nighttime (stable conditions) LST warming, while daytime (unstable conditions) effects remain inconclusive. However, two key distinctions differentiate SST warming from LST warming. First, while both offshore and onshore wind farms generate anomalous turbulent mixing in the atmosphere, which transports warmer air downward and increases near-surface air temperatures, the mechanisms driving surface temperature changes differ. On shore, LST warming is primarily linked to changes in surface turbulent heat fluxes resulting from changes in turbulent mixing (*61*). Offshore, SST warming is controlled more by changes in ocean mixed layer stratification, turbulent mixing, and upwelling circulation driven by changes in wind stress (*13*, *16*). The resultant SST increase, in turn, drives anomalous ocean-to-atmosphere heat fluxes. Without the ocean acting as an anomalous heat source, the increased upward heat flux changes would promote SST cooling. Second, while LST changes may be sensitive to atmospheric stability and turbulence parameters, the SST warming in our simulations exhibits only a weak dependence on these parameters (Fig. 7). These differences suggest that upper-ocean processes may play an important role in shaping SST responses to offshore wind farms.

The wake-induced ocean effects reported here are likely modulated by factors not addressed in this study, such as wind farm layout, installed capacity, development scale, and turbine specifications (*6*, *49*). Our results, therefore, motivate future sensitivity experiments to assess how different design and operational choices may influence atmospheric and oceanic responses as well as energy output. Such efforts can support optimized wind farm layouts that reduce energy losses and minimize the levelized costs of energy (*5*, *62*) while mitigating impacts on the ocean and ecosystems (*8*).

Moreover, while this study evaluated the sensitivity of SST responses under different stability conditions and turbulence parameters, additional uncertainty quantification is necessary. Given the important role of turbulent boundary layer processes in shaping modeled SST and mixed layer dynamics, future work should explore alternative turbulence closure approaches, such as the three-dimensional PBL scheme in the atmosphere (*63*) and various vertical mixing schemes for the ocean surface layer. These parameterizations represent different turbulence physics governing vertical mixing and entrainment (*64*, *65*) and may influence the evolution of simulated SST and MLD responses. To better understand the nonlinear behavior of these processes, ensemble-based diagnostic tools—such as ensemble vector methodologies (*65*)—can be used in future studies to isolate model sensitivities and quantify feedback mechanisms arising from perturbations in coupled systems.

In conclusion, this study contributes to our understanding of how turbine-induced wind wakes may influence coupled ocean-atmosphere processes. Our simulations suggest that two-way air-sea interactions can play a role in shaping near-surface ocean and atmospheric conditions in the vicinity of offshore wind farms. By examining the physical mechanisms involved, this work helps clarify the circumstances under which ocean-atmosphere coupling may be necessary for meteorological, oceanographic, and wind energy modeling. As interest in the broader oceanographic, ecological, and atmospheric implications of offshore wind development continues to grow, these findings support the potential value of incorporating coupled air-sea interaction models into future environmental assessments.

## MATERIALS AND METHODS

### Ocean-atmosphere-wave coupled model

This study uses the Scripps Coupled Ocean-Atmosphere Regional (SCOAR) modeling system, which integrates the Weather Research and Forecasting (WRF) model (*66*) for the atmosphere, the Regional Ocean Modeling System (ROMS) (*67*) for the ocean, and the third-generation spectral wave model WaveWatch III (WW3) (*68*) for surface waves. Detailed model coupling procedures are discussed in (*69*, *70*). Briefly, air-sea momentum, turbulent heat, and freshwater fluxes are computed using the Coupled Ocean-Atmosphere Response Experiment (COARE) wave–based bulk flux parameterization (*71*, *72*), which is implemented in the WRF surface layer scheme (*73*). This parameterization includes modifications to account for sea state impact on air-sea momentum fluxes (*72*). Along with the standard meteorological outputs from WRF and the SST and surface currents from ROMS, the COARE wave–based formulations incorporate the dominant wave phase speed and significant wave height from WW3 to parameterize wave-supported roughness length (*70*, *74*). A complete list of the physical parameterizations used for this study is provided in table S2.

### Experimental setup

WRF uses a one-way nesting approach with a horizontal grid spacing of 1.5 km in the inner (nested) domain (Fig. 1A) to capture fine-scale coupled ocean-atmosphere interactions. This domain is driven by the large-scale atmospheric circulation from the outer domain with a 7.5-km grid spacing, which dynamically downscales the European Centre for Medium-Range Weather Forecasts Reanalysis V5 (ERA5) (*75*). Spectral nudging is applied in the outer domain above the planetary boundary layer, constraining zonal and meridional wavelengths longer than 850 and 730 km, respectively, to maintain consistency with the ERA5 free tropospheric circulation. No spectral nudging is applied in the nested domain, which spans the Mid-Atlantic Bight region.

The WRF, ROMS, and WW3 models are coupled hourly in the nested domain, using identical grids and land-sea masks. The WRF vertical grid consists of 50 levels, with 20 below 250 m. The lowest model level is positioned at 5.5 m and the second at 12 m, enabling improved representation of near-surface processes, especially under stable conditions (*43*). The mesh dimensions are 228 by 195 by 50 (nx, ny, and nz) in the outer WRF-only domain (d01) and 495 by 330 by 50 in the nested, fully coupled domain.

ROMS uses a 30-level stretched vertical grid, with higher resolution near the ocean surface and bottom. Its grid dimensions are 495 by 330 by 30 (nx, ny, and nz). WW3 uses the same horizontal grid dimensions of 495 by 330 (nx and ny) and is configured with 32 frequency bins and 24 directional bins. Both ROMS and WW3 use the same bathymetric data from the General Bathymetry Chart of the Oceans at 1′ grid spacing (*76*). In ROMS, the minimum ocean depth is set to 10 m, bottom friction is represented using quadratic drag, and vertical grid parameters are defined as $\theta_{s}$ = 7, $\theta_{b}$ = 2, and $h_{\text{cline}}$ = 300 m.

WRF is initialized and forced at the lateral boundaries with hourly 0.25° ERA5 reanalysis (*75*). ROMS is initialized and forced using the daily 1/12° MERCATOR International global reanalysis (*77*). WW3 is forced by global 0.5° WW3 simulations with 14 wave spectral points (*78*) and is initialized from the end of a 30-day spin-up simulation for each year, driven by ERA5 winds.

ROMS tidal forcing is generated using the Oregon State University Tidal Prediction Software (*79*), providing boundary conditions for 13 major tidal constituents. These include tidal periods, sea surface elevation amplitudes, current phases, inclination angles, and ellipse semiminor axes, as implemented in (*80*).

### Wind farm parameterization

Wind turbine wake effects are modeled using the Fitch parameterization (*1*), as implemented in WRF v4.2.2, including the correction noted by Archer *et al.* (*33*). The scheme represents wind turbines as sinks of momentum and sources of TKE at the model levels that intersect the rotor-swept areas. Advection of TKE is enabled. The TKE generated by the turbines is not calculated internally in the Fitch scheme but is instead specified via an empirical parameter, α (*33*). The default value α = 0.25 indicates that 25% of the turbine-generated TKE is introduced into the atmospheric model. Previous studies have explored a range of α values to account for different assumptions about turbine-induced turbulence (*10*, *20*, *22*, *33*, *34*). Given the sensitivity of near-surface wind simulations to α, this study examines two configurations: α = 0.25 and α = 1.0 (table S1).

The turbine dimensions, power and thrust coefficients, and layout configurations used in this study follow those described by Rosencrans *et al.* (*20*). A total of 1418 wind turbines are placed across the Mid-Atlantic Bight lease areas (Fig. 1A). Each turbine has a rated capacity of 12 MW, a hub height of 138 m, and a rotor diameter of 215 m. This configuration positions the bottom and top blade tips at 30.5 and 245.5 m, respectively. In WRF, 15 vertical model levels intersect the rotor-swept area, with additional four levels below the lowest blade tip. The fine vertical resolution is critical for accurately capturing turbine wake effects in the atmospheric boundary layer (*43*).

### Experiments

A series of experiments is conducted to evaluate the effects of offshore wind farms and ocean-atmosphere coupling (table S1). The simulation labeled OC_WF includes both ocean coupling (OC) and wind farms (WF); OC_NWF includes ocean coupling but no wind farms, serving as the unperturbed reference case; and NOC_WF excludes ocean coupling but includes wind farms. Both OC_WF and OC_NWF are fully coupled simulations, while NOC_WF is a WRF-only simulation forced with a blended SST field.

In the NOC_WF setup, the SST field is identical to that from OC_WF except in regions with statistically significant positive SST responses (Fig. 3A). In those regions, at every coupling time step, SSTs from OC_NWF are applied to suppress the wake-induced ocean warming. The spatial mask for blending is based on the time-invariant extent of significant SST anomalies. To ensure a smooth transition between blended SST fields, a linear taper is applied across the boundary zones. This experimental design isolates the effect of wind farm-induced SST anomalies, ensuring that the only difference between OC_WF and NOC_WF is the presence or absence of the coupled ocean response to wakes.

All simulations span the summer months [June to August (JJA)] over 5 consecutive years (2017–2021). Wind wake effects are generally more pronounced during summer due to frequent stable atmospheric stratification in this region (*5*, *20*, *29*, *30*, *81*). Nonetheless, unstable conditions occurred during ~20 to 37% of the study period in the observations and simulations (fig. S13), necessitating a stability-dependent analysis of wind farm effects. Simulations were averaged over the five summers to characterize the time-mean wake effects. While longer simulations could improve statistical robustness, the simulated wake characteristics agree well with those from previous year-long studies (*10*, *20*).

The unperturbed case, OC_NWF, reproduces key statistics of the near-surface wind field, including wind direction and magnitude (Fig. 1B), as well as the observed stability-dependent variability (fig. S13). It also captures summertime surface wave statistics, including significant wave height and direction of the dominant wave, consistent with buoy observations (fig. S14). These comparisons demonstrate that our unperturbed simulation represents the summertime near-surface ocean-atmosphere processes critical to this study.

### Statistical significance and adjusted degrees of freedom

A two-sided Student’s *t* test was used to evaluate the statistical significance of time-averaged differences between simulations. The effective sample size, *n′*, accounting for temporal autocorrelation within each time series, is computed following Bretherton *et al.* (*82*)

$$n^{\prime }=\frac{n}{\sum_{\tau =-\left(n-1\right)}^{n-1}\left(1\;-\;|\;\tau \;|/n\right)\rho_{\tau }^{2}}$$ (1)

Here, *n* is the daily sample size (*n* = 276), and $\rho_{\tau }$ is the time-series autocorrelation at lag τ. Since the time series is segmented by year, *n′* is computed separately for each summer and then summed to obtain a final *n′* for the entire period. Statistical significance is assigned only when the final *P* value indicates significance at the 99% confidence level (*P* < 0.01). This significance criterion also determines the spatial extent of warm SST anomalies to construct the SST forcing for the NOC_WF run.

### Atmospheric stability classification

Atmospheric stability is determined by the Obukhov length (*83*)

$$L=-\frac{u_{*}^{3}\bar{\theta_{v}}}{\mathrm{\kappa g}\left(\bar{w^{\prime }\theta_{v}^{\prime }}\right)}$$ (2)

Here, $u_{*}$ is the friction velocity, $\theta_{v}$ is the virtual potential temperature, κ is the von Kármán constant (0.4), *g* is the gravitational acceleration, and $\bar{w^{\prime }\theta_{v}^{\prime }}$ is the vertical turbulent heat flux. *L* is estimated as the reciprocal of the hourly WRF output variable (RMOL), which can differ from *L* calculated from fluxes about 6% of the time (*84*). The atmosphere is classified as stably stratified when 0 m < *L* < 500 m, unstably stratified when −500 m < *L* < 0 m and neutrally stratified when ∣L∣> 500 m following (*20*). When averaged over the MA/RI lease areas, stable conditions occur ~59.3% of the hourly data, unstable conditions 35.6%, and neutral conditions 5.1% in the unperturbed case (OC_NWF). The stability-dependent composite averages presented in this study are based on *L* over the MA/RI lease areas. However, we find that similar results are obtained even if using *L* calculated over the NJ lease areas.

### Ocean mixed layer heat budget

The vertically averaged MLT budget equation is derived from the conservation of mass and heat, such that

$$\begin{array}{c} \begin{array}{c} \underset{\text{TEND}}{\underbrace{\frac{\partial \langle T\rangle }{\partial \;t}}}=\\ \underset{\text{HADV}}{\underbrace{-\langle \nabla \cdot \left(uT\right)\rangle }}\underset{\text{VADV}}{\underbrace{+\frac{1}{H}{\left(\mathrm{wT}\right)}_{z=-h}}}\underset{\text{HMIX}}{\underbrace{+\langle \nabla \cdot \left(\kappa_{h}\nabla T\right)\rangle }}\underset{\text{VMIX}}{\underbrace{-\frac{1}{H}{\left(\kappa_{v}\frac{\partial \;T}{\partial \;z}\right)}_{z=-h}}}\underset{\text{ENT}}{\underbrace{-\frac{\Delta T}{H}\frac{\partial \;h}{\partial \;t}}}\underset{\text{QFLX}}{\underbrace{+\frac{{Q_{\text{net}}-Q_{\text{sw}}|}_{z=-h}}{\rho c_{p}H}}} \end{array} \end{array}$$ (3)

Here, *T* is the MLT, *h* is the MLD, and the angle brackets indicate vertical averaging over H=h+η , where η is the sea surface height. ROMS discretizes the tracer (e.g., temperature) evolution equations in conservation form, meaning that advection terms are expressed as the divergence of tracer fluxes. The MLT tendency (TEND) of Eq. 3 is balanced by the terms on the right-hand side.

HADV and VADV describe divergence of heat flux due to advection by the horizontal current, ***u*** = (*u*, *v*), and vertical transport through the base of the mixed layer. *w* is the vertical velocity. Note that wT=0 at the sea surface. HMIX and VMIX correspond to horizontal and vertical turbulent mixing, with $\kappa_{h}$ and $\kappa_{v}$ denoting the horizontal and vertical diffusivity coefficients, respectively. HMIX, representing horizontal mixing or diffusion, is generally negligible relative to other terms. ENT captures entrainment cooling, where ΔT is the temperature difference between the mixed layer and the layer below. This term quantifies the cooling effect of incorporating colder subsurface water resulting from the deepening of the MLD. The term QFLX represents the surface heat flux, where $Q_{\text{net}}$ is net heat flux (positive when heating the ocean), and ${Q_{\text{sw}}|}_{z=-h}$ is the portion of shortwave radiation that penetrates below the mixed layer, hence does not contribute to the MLT budget. ρ and $c_{p}$ are seawater density and the specific heat capacity, respectively.

We computed each term in Eq. 3 using daily averaged ROMS output. With online temperature diagnostics enabled, ROMS calculates temperature tendencies from each term in the tracer equation at every model level and time step, ensuring closure of the mixed-layer heat budget. Consequently, the HADV, VADV, HMIX, and VMIX terms were evaluated by vertically averaging their contributions over the depth, *H*, weighted by the layer thickness. The ENT term was computed using the method described by Kim *et al.* (*85*), which ensures budget closure over the averaging interval. The closure is achieved by explicitly accounting for relatively colder water that is removed during mixed layer shoaling (detrainment), leading to an increase in MLT. To estimate the QFLX term, we calculated the attenuation of shortwave radiation using Jerlov coastal water type (type II) to determine ${Q_{\text{sw}}|}_{z=-h}$ . The time-averaged budget terms in Eq. 3 (in units of °C per day) for the unperturbed simulation are shown in fig. S10. To compare contributions between simulations, each term is integrated over time to yield MLT equivalents (in units of °C), as shown in Fig. 8 and fig. S11. These MLT equivalents are then averaged over corresponding control volumes, defined by regions of warm SST anomalies near the MA/RI and NJ wind farms, to evaluate the volume-averaged contribution of each budget term.

## Supplementary Materials

### This PDF file includes:

Figs. S1 to S14 Tables S1 to S3 References
