# Supplementary material for: Sea surface warming and ocean-to-atmosphere feedback driven by large-scale offshore wind farms under seasonally stratified conditions
Source: Sci Adv. 2025 Nov 5;11(45):eadw7603. doi: 10.1126/sciadv.adw7603 (PMC12588259; doi:10.1126/sciadv.adw7603)
Supplement: Supplementary file 1 — Figs. S1 to S14 Tables S1 to S3 References [file sciadv.adw7603_sm.pdf]

Science Advances
AAAS

Supplementary Materials for

Sea surface warming and ocean-to-atmosphere feedback driven by large-scale offshore wind farms under seasonally stratified conditions

Hyodae Seo *et al.*

Corresponding author: Hyodae Seo, hyodae@hawaii.edu

Sci. Adv. **11**, eadw7603 (2025)
DOI: 10.1126/sciadv.adw7603

This PDF file includes:

Figs. S1 to S14
Tables S1 to S3
References

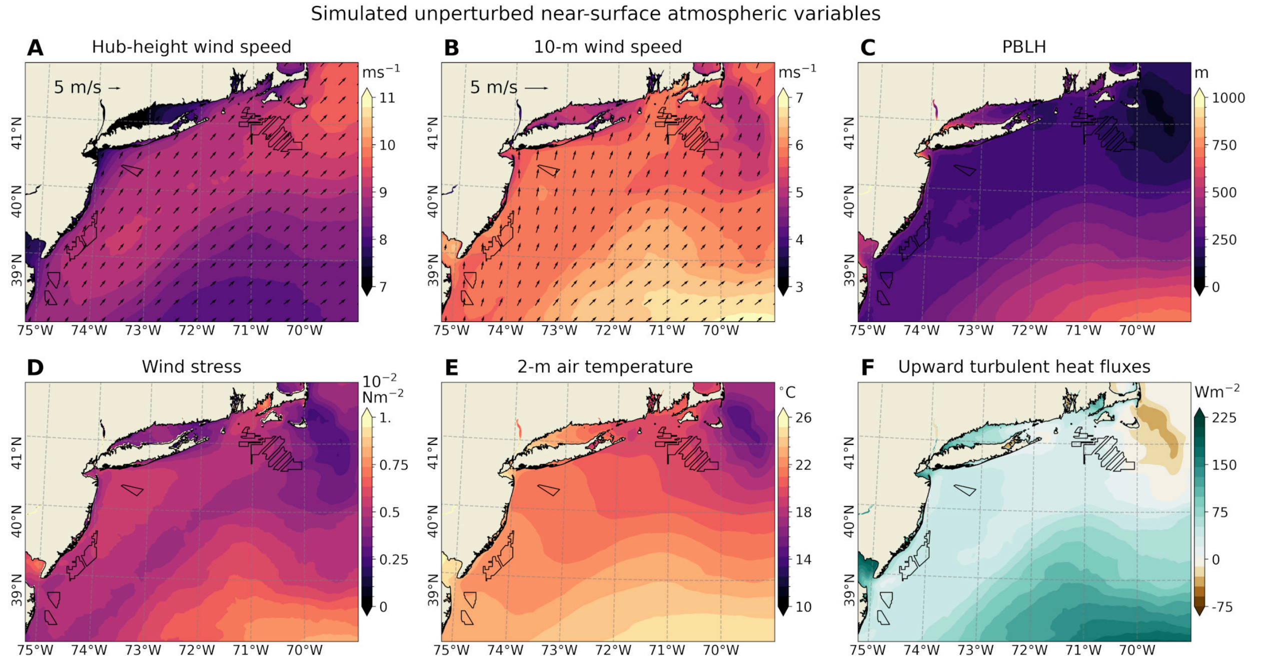

Figure S1: **Simulated Unperturbed Near-Surface Atmospheric Variables.** Similar to Figure 2, but showing results from the unperturbed simulation (OC_NWF).

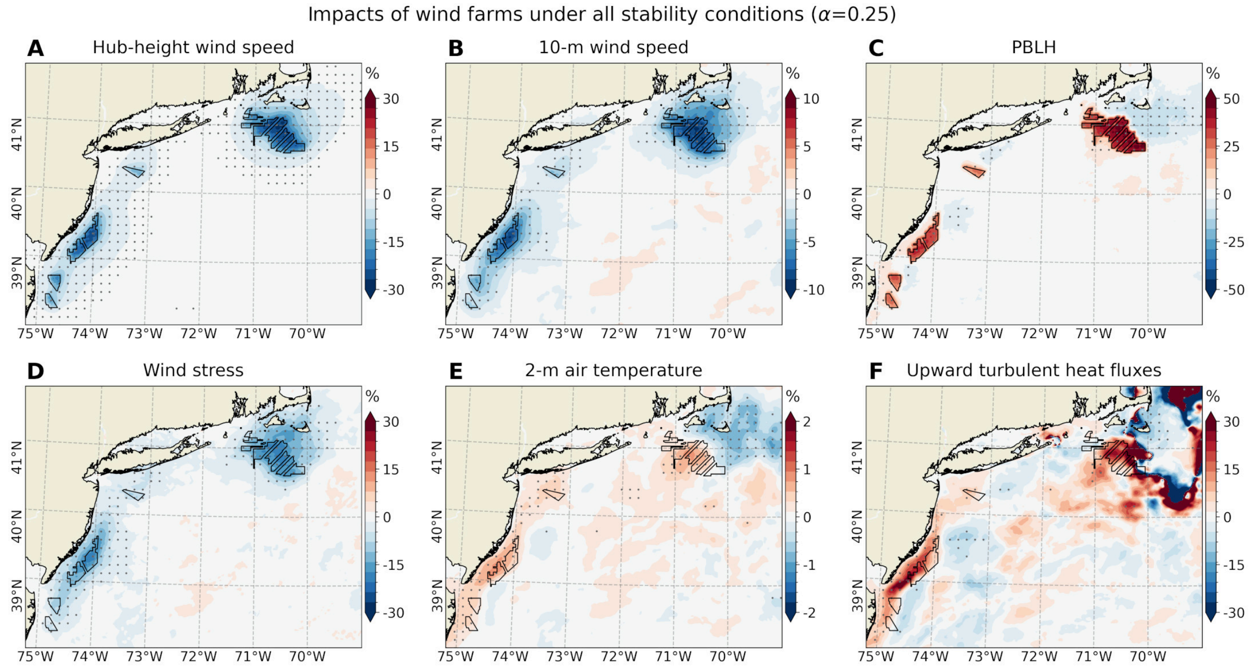

Figure S2: **Impacts of Wind Farms Under ALL Stability Conditions** (α**=**0.25)**.** Similar to Figure 2, but with the differences expressed as percentage changes relative to OC_NWF, calculated as $^{((\text{OC\_WF}-\text{OC\_NWF})}/_{\text{OC\_NWF}})\times 100\%$. The noisy changes in (F) are due to small climatological heat flux values in OC_NWF in such regions (Figure S1f).

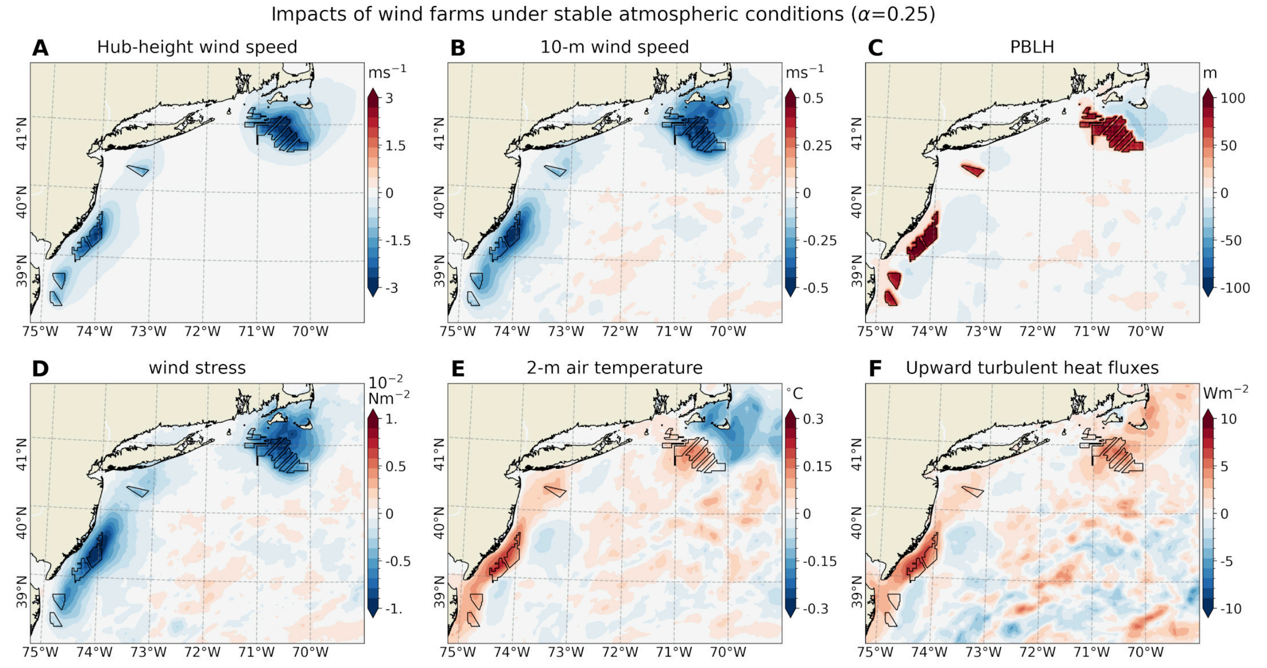

Figure S3: **Impacts of Wind Farms Under Stable Atmospheric Conditions** (α**=**0.25)**.** Similar to Figure 2, but showing averages for stable atmospheric conditions only (58.6% of the hourly data).

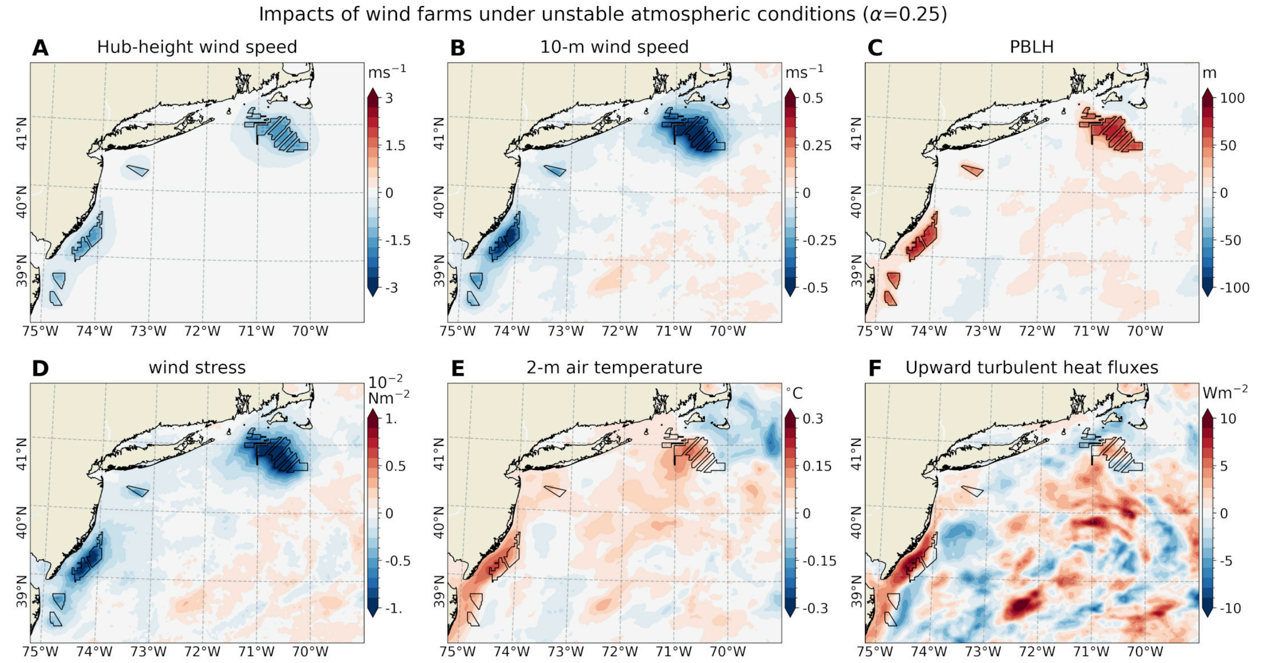

Figure S4: **Impacts of Wind Farms Under Unstable Atmospheric Conditions** (α**=**0.25)**.** Similar to Figure 2, but showing averages for unstable atmospheric conditions only (36% of the hourly data).

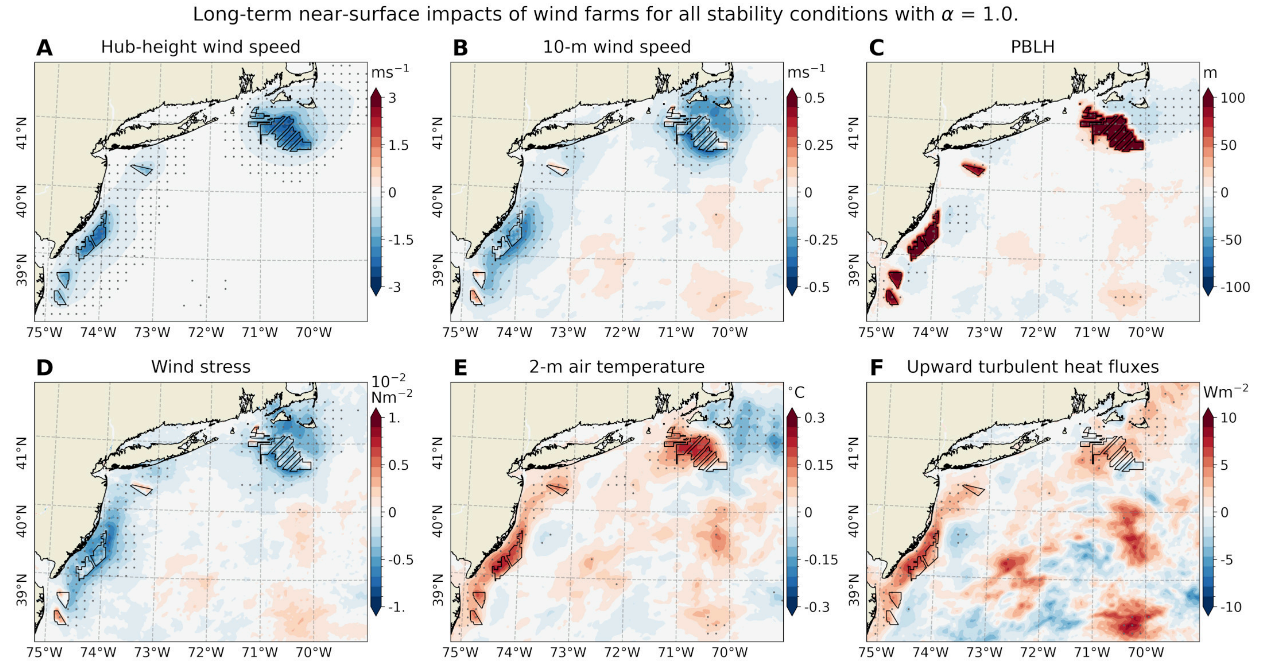

Figure S5: **Impacts of Wind Farms Across All Stability Conditions** (α**=**1.0)**.** Similar to Figure 2, but simulated with α=1.0.

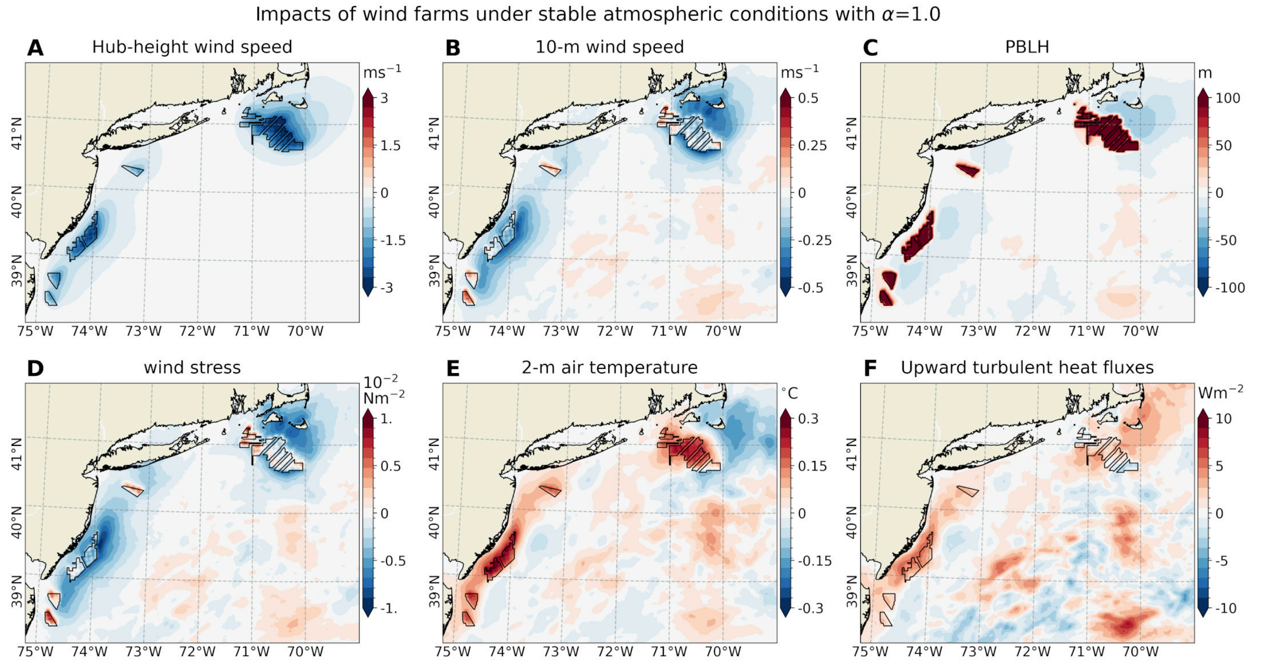

Figure S6: **Impacts of Wind Farms Under Stable Atmospheric Conditions** (α**=**1.0)**.** Similar to Figure S3, but simulated with α=1.0.

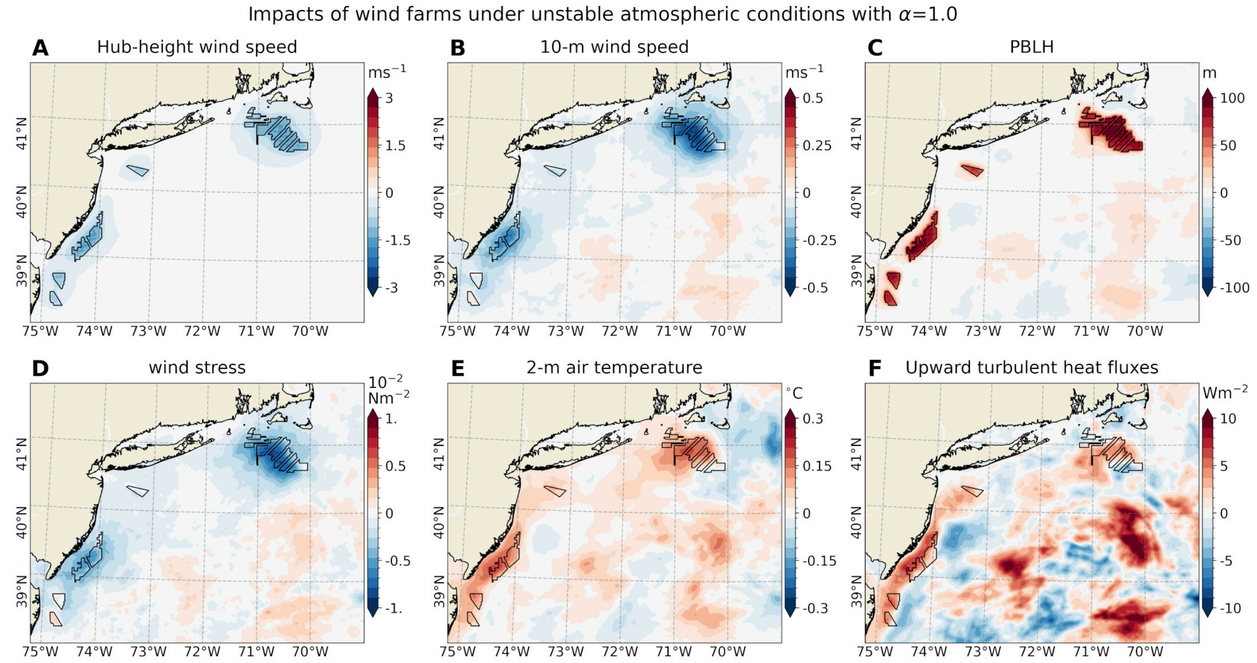

Figure S7: **Impacts of Wind Farms Under Unstable Atmospheric Conditions** (α**=**1.0)**.** Similar to Figure S4, but simulated with α=1.0.

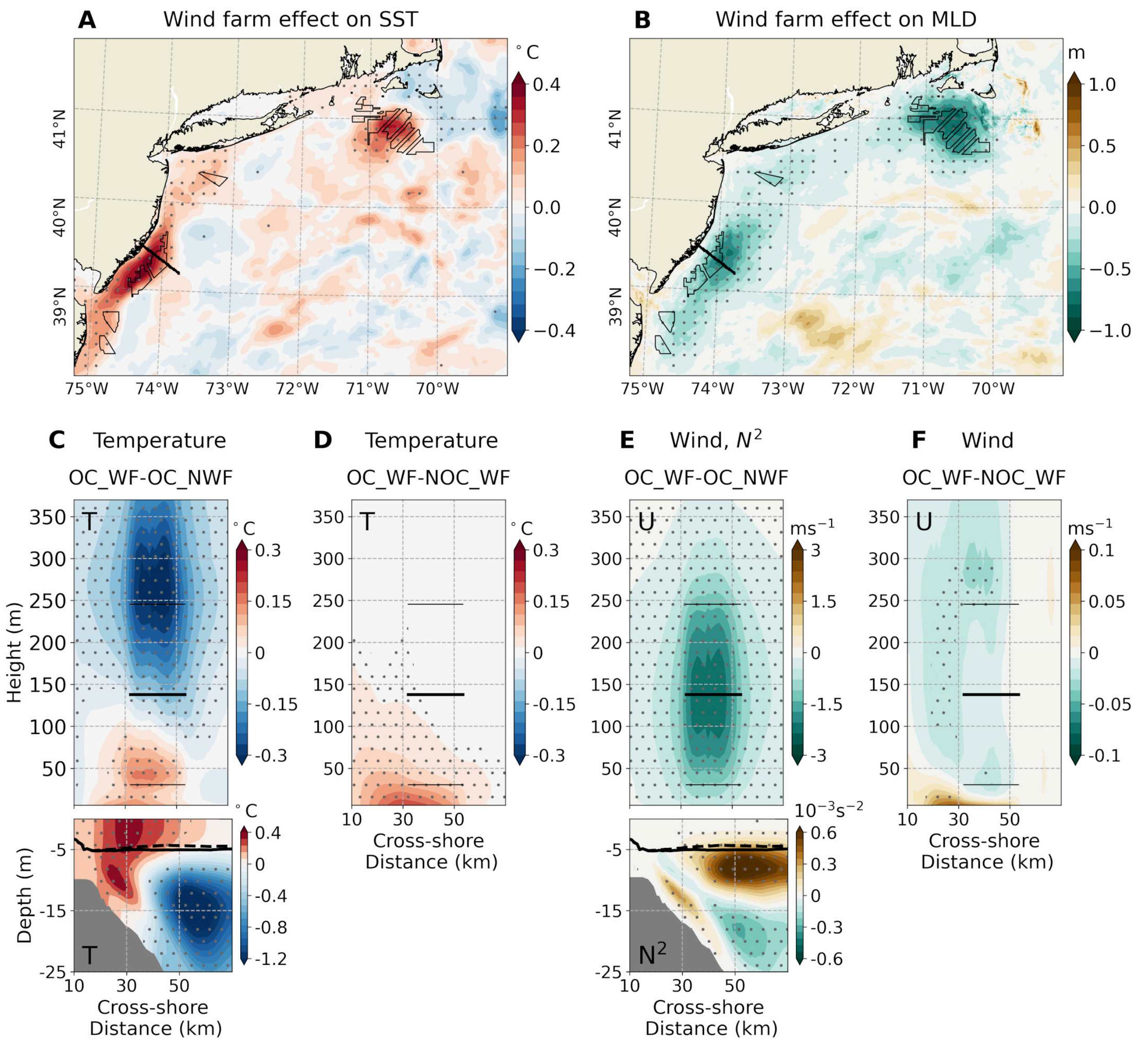

Figure S8: **Impacts of Wind Farms on Vertical Ocean-Atmosphere Structure: NJ focus.** Similar to Figure 3, but for the New Jersey (NJ) cross sections, with the x-axis representing the distance from the shore in kilometers. Results are averaged across all atmospheric stability conditions and simulated with α=0.25.

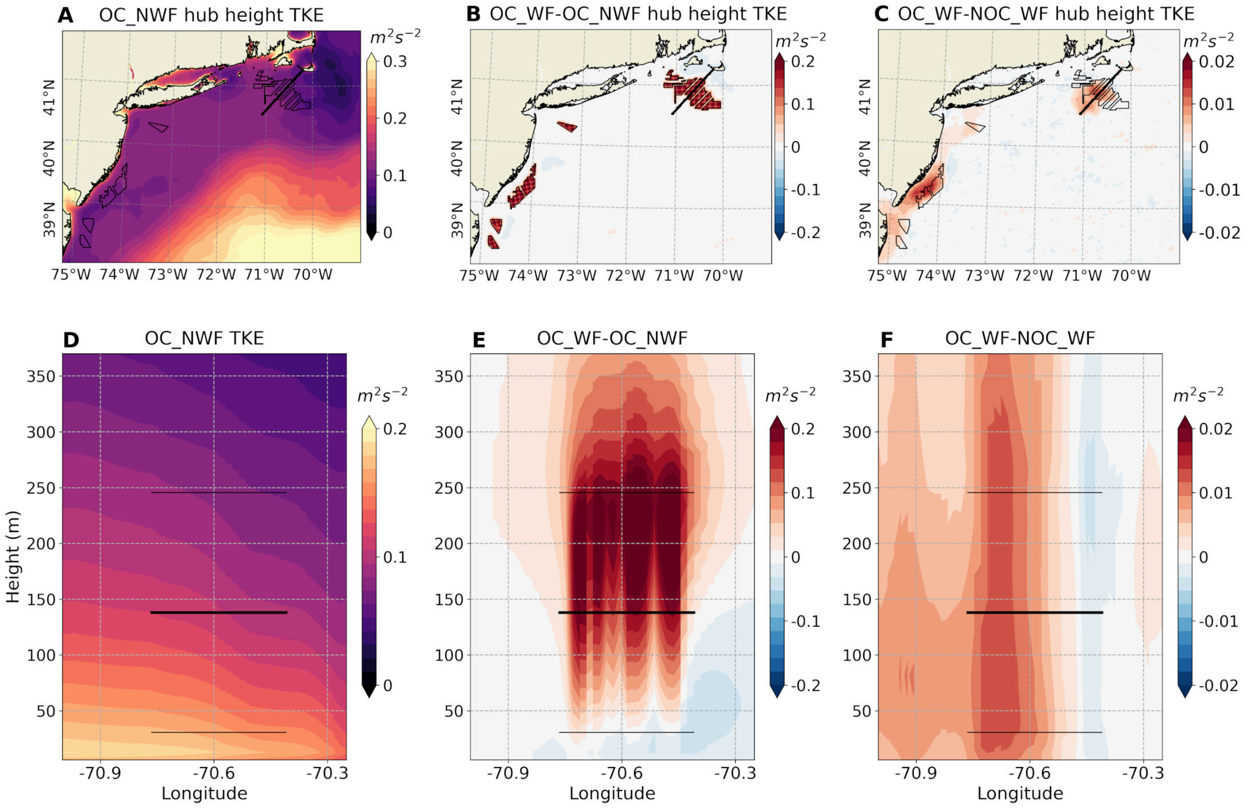

Figure S9: **Changes in TKE in the MABL due to wind farms and ocean coupling.** Maps and cross-sections of TKE from (A,D) unperturbed simulation, and differences (B,E) due to wind farms (OC_WF–OC_NWF) and (C,F) due to ocean coupling (OC_WF–NOC_WF).

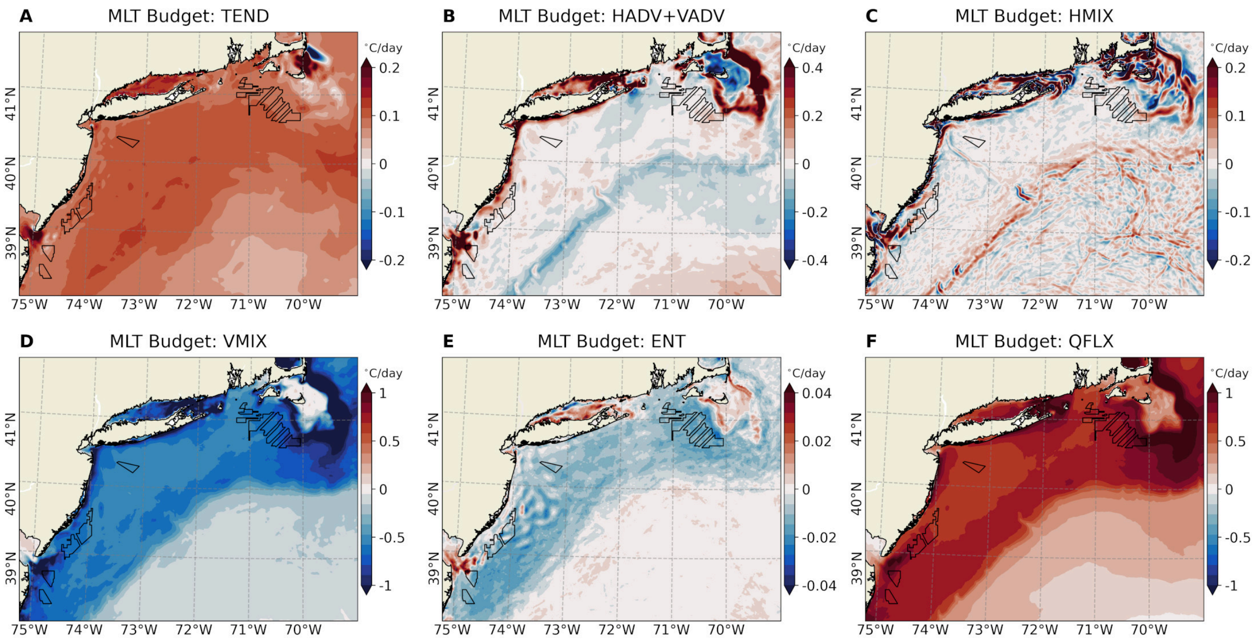

Figure S10: **Mixed Layer Temperature Balance in the Unperturbed Simulations.** Time-averaged (JJA, 2017–2021) MLT tendency ($°\mathrm{C\,d^{-1}}$) associated with each term in the MLT budget equation (Eq. 3). Note the different color scales in each panel.

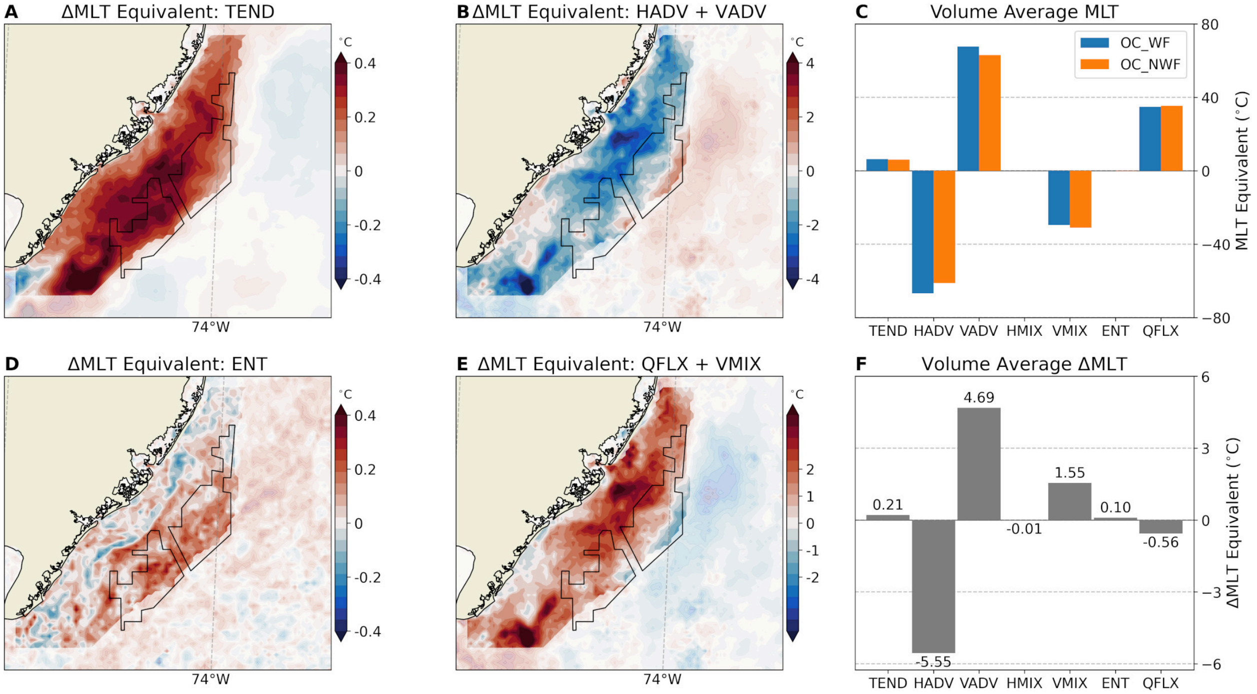

Figure S11: **Changes in Mixed Layer Temperature Equivalents in NJ.** As in Figure 8, but for the NJ coast. Note the different color scales in A, B, D, and E.

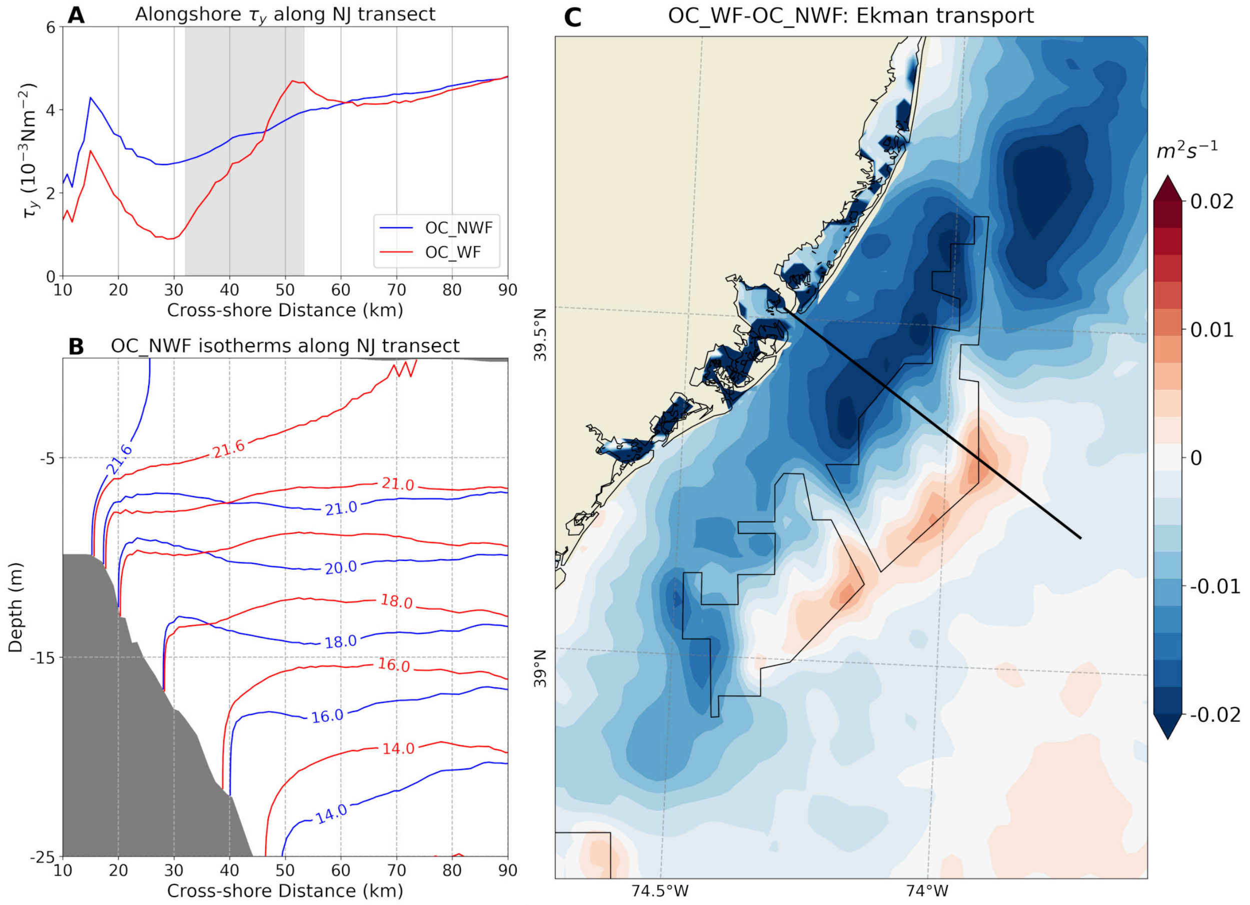

Figure S12: **Changes in cross-shore Ekman Transport along the NJ coast.** (A) Cross-shelf section (along the black line in panel (C)) of along-shore wind stress, and (B) isotherms from OC_NWF (blue) and OC_WF (red). (C) Map of changes in cross-shore Ekman transport (m^2s^{-1}) driven by differences in along-shore wind stress. Ekman transport is calculated as $\tau_y/(\rho_o f)$, where τ_y is the along-shore wind stress, ρ_o is the reference seawater density, and f is the Coriolis parameter. Gray shading in (A) indicates the location of NJ lease areas.

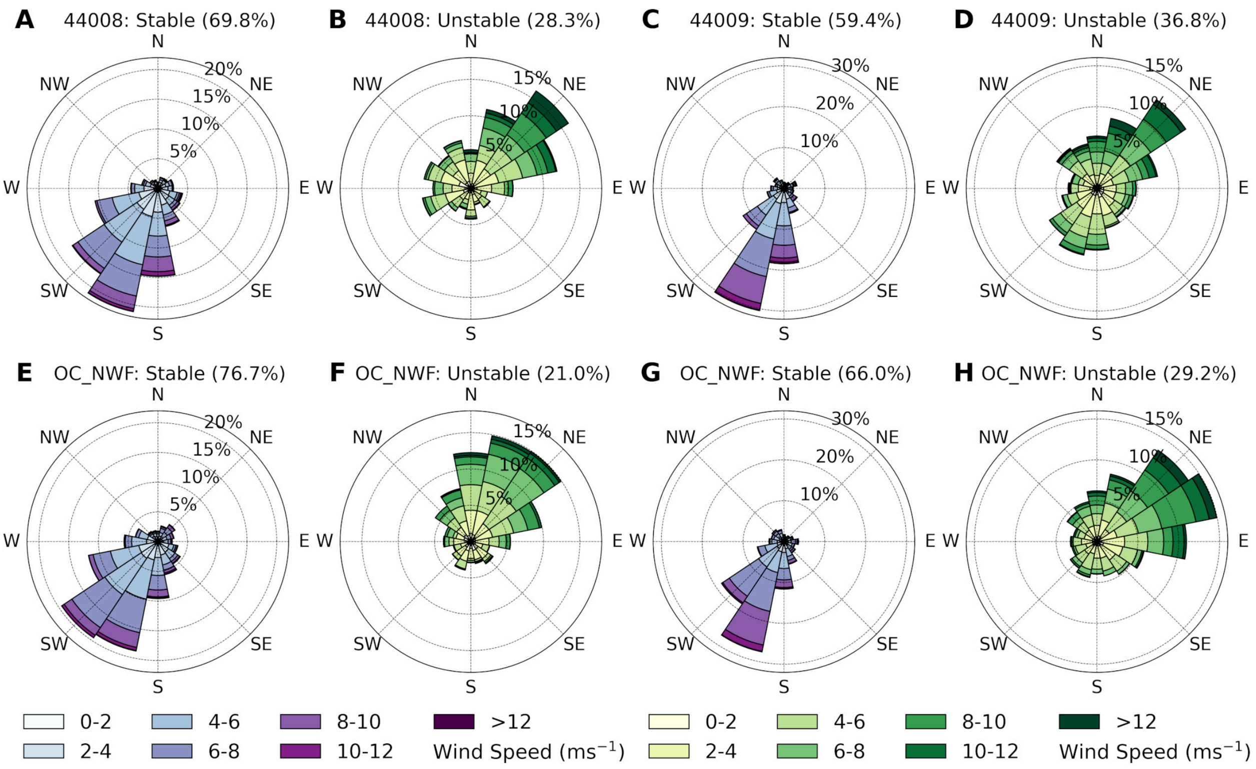

Figure S13: **Observed and Simulated Winds Under Different Atmospheric Stability Conditions in the Unperturbed Simulation.** (Top) Wind roses showing observed wind direction (origin) and wind speed (shading) under stable (blue) and unstable (green) conditions at (A,B) NDBC Buoy 44008 and (C,D) NDBC Buoy 44009. (Bottom) Corresponding wind roses from the unperturbed simulation (OC_NWF). Atmospheric stability in the buoy observations is defined using SST-T2 (positive values indicate unstable conditions), as the Monin-Obukhov length (L) cannot be calculated from buoy data. Model-based stability classifications using L yield similar wind direction, magnitude, and frequency distributions compared to those based on SST–T2.

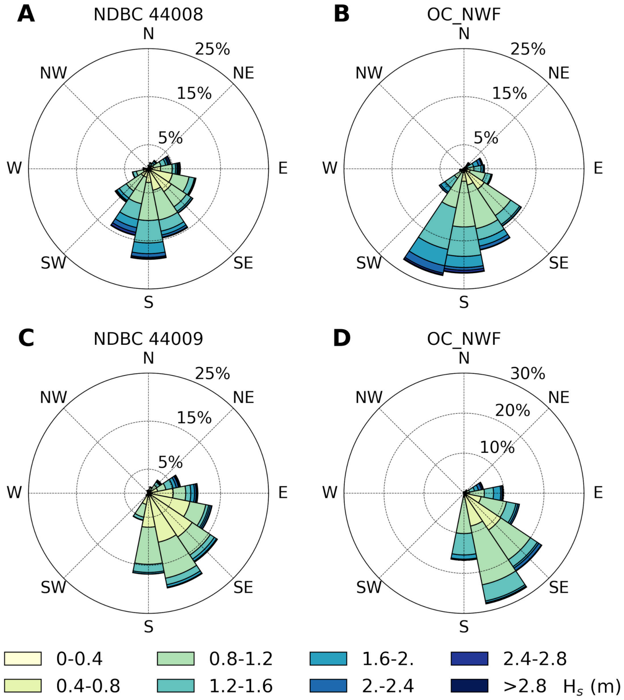

Figure S14: **Observed and Simulated Waves Across All Stability Conditions.** (Left) Wave roses showing observed dominant wave direction (origin) and significant wave height (H_s, m, shading) at NDBC buoys 44008 and 44009 (JJA, 2017–2021). (Right) Corresponding wave roses from the unperturbed model simulation (OC_NWF).

Table S1: Summary of model experiments.

Experiments	Wind Farms (WF)	Ocean Coupling (OC)	Number of turbines	TKE factor, α
OC_WF	Yes	Yes	1418	0.25
OC_NWF	No	Yes	0	0.25
NOC_WF	Yes	No	1418	0.25
OC_WF100	Yes	Yes	1418	1.00

Table S2: **Model physics**

Model	Physics	Scheme
WRF	Deep convection	Kain-Fritsch (*87*)
	Cloud microphysics	Thompson (*88*)
	Land surface	Noah land surface model (*89*)
	Shortwave/longwave radiation	Rapid Radiative Transfer Model (*90*)
	Planetary boundary layer	Mellor-Yamada-Nakanishi-Niino (MYNN) level 2.5 (*91*)
	Surface layer	MYNN (*73*)
ROMS	Vertical mixing	Generic Length Scale k–ϵ scheme (*92*)
	Stability function	Canuto-B (*93*)
	Horizontal advection	3rd-order upstream-biased (*67*)
	Vertical advection	4th-order centered differences (*67*)
WW3	Wind input and wave dissipation	Source Term (ST4) Package (*94, 95*)
	Wave reflection	Enables reflection of shorelines (*96*)
	Depth-induced wave breaking	Battjes-Janssen model (*97*)
	Bottom friction	SHOWEX bottom friction formulation (*98*)

Table S3: **Accumulated power over the study period (JJA, 2017–2021) across grid cells containing turbines within the MA/RI and NJ lease areas.**

	OC_WF (MWh)	NOC_WF (MWh)	OC_WF - NOC_WF (MWh)	OC_WF - NOC_WF (%)
MA/RI	47,367.6	47,428.4	-60.9	-0.13
NJ	47,697.9	47,814.2	-116.4	-0.24

REFERENCES AND NOTES

1. A. C. Fitch, J. B. Olson, J. K. Lundquist, J. Dudhia, A. K. Gupta, J. Michalakes, I. Barstad, Local and mesoscale impacts of wind farms as parameterized in a mesoscale NWP model. *Mon. Weather Rev.* **140**, 3017–3038 (2012).

2. P. J. H. Volker, J. Badger, A. N. Hahmann, S. Ott, The explicit wake parametrisation V1.0: A wind farm parametrisation in the mesoscale model WRF. *Geosci. Model Dev.* **8**, 3715–3731 (2015).

3. N. Akhtar, B. Geyer, B. Rockel, P. S. Sommer, C. Schrum, Accelerating deployment of offshore wind energy alter wind climate and reduce future power generation potentials. *Sci. Rep.* **11**, 11826 (2021).

4. J. Fischereit, R. Brown, X. G. Larsén, J. Badger, G. Hawkes, Review of mesoscale wind-farm parametrizations and their applications. *Bound. Layer Meteorol.* **182**, 175–224 (2022).

5. S. C. Pryor, R. J. Barthelmie, T. J. Shepherd, Wind power production from very large offshore wind farms. *Joule* **5**, 2663–2686 (2021).

6. M. Golbazi, C. L. Archer, S. Alessandrini, Surface impacts of large offshore wind farms. *Environ. Res. Lett.* **17**, 064021 (2022).

7. N. Akhtar, B. Geyer, C. Schrum, Impacts of accelerating deployment of offshore windfarms on near-surface climate. *Sci. Rep.* **12**, 18307 (2022).

8. N. Akhtar, B. Geyer, C. Schrum, Larger wind turbines as a solution to reduce environmental impacts. *Sci. Rep.* **14**, 6608 (2024).

9. X. G. Larsén, J. Fischereit, A case study of wind farm effects using two wake parameterizations in the Weather Research and Forecasting (WRF) model (V3.7.1) in the presence of low-level jets. *Geosci. Model Dev.* **14**, 3141–3158 (2021).

10. D. Quint, J. K. Lundquist, N. Bodini, D. Rosencrans, Simulated meteorological impacts of offshore wind turbines and sensitivity to the amount of added turbulence kinetic energy. *Wind Energy Sci.* **10**, 1269–1301 (2025).

11. I. Galparsoro, I. Menchaca, J. M. Garmendia, A. Borja, A. D. Maldonado, G. Iglesias, J. Bald, Reviewing the ecological impacts of offshore wind farms. *NPJ Ocean Sustain.* **1**, 1 (2022).

12. National Academies of Sciences, Engineering, and Medicine, *Potential Hydrodynamic Impacts of Offshore Wind Energy on Nantucket Shoals Regional Ecology: An Evaluation from Wind to Whales* (National Academies Press, 2024).

13. N. Christiansen, U. Daewel, B. Djath, C. Schrum, Emergence of large-scale hydrodynamic structures due to atmospheric offshore wind farm wakes. *Front. Mar. Sci.* **9**, 818501 (2022).

14. U. Daewel, N. Akhtar, N. Christiansen, C. Schrum, Offshore wind farms are projected to impact primary production and bottom water deoxygenation in the North Sea. *Commun. Earth Environ.* **3**, 292 (2022).

15. K. Raghukumar, C. Chartrand, G. Chang, L. Cheung, J. Roberts, Effect of floating offshore wind turbines on atmospheric circulation in California. *Front. Energy Res.* **10**, 863995 (2022).

16. K. Raghukumar, T. Nelson, M. Jacox, C. Chartrand, J. Fiechter, G. Chang, L. Cheung, J. Roberts, Projected cross-shore changes in upwelling induced by offshore wind farm development along the California coast. *Commun. Earth Environ.* **4**, 116 (2023).

17. T. Miles, S. Murphy, J. Kohut, S. Borsetti, D. Munroe, Offshore wind energy and the mid-atlantic cold pool: A review of potential interactions. *Mar. Technol. Soc. J* **55**, 72–87 (2021).

18. S. J. Lentz, Seasonal warming of the middle atlantic bight cold pool. *J. Geophys. Res. Oceans* **122**, 941–954 (2017).

19. G. Xia, M. C. Cervarich, S. B. Roy, L. Zhou, J. R. Minder, P. A. Jimenez, J. M. Freedman, Simulating impacts of real-world wind farms on land surface temperature using the WRF model: Validation with observations. *Mon. Weather Rev.* **145**, 4813–4836 (2017).

20. D. Rosencrans, J. K. Lundquist, M. Optis, A. Rybchuk, N. Bodini, M. Rossol, Seasonal variability of wake impacts on US mid-Atlantic offshore wind plant power production. *Wind Energy Sci.* **9**, 555–583 (2024).

21. J. Fischereit, X. G. Larsén, A. N. Hahmann, Climatic impacts of wind-wave-wake interactions in offshore wind farms. *Front. Energy Res.* **10**, 881459 (2022).

22. X. G. Larsén, J. Fischereit, S. Hamzeloo, K. Bärfuss, A. Lampert, Investigation of wind farm impacts on surface waves using coupled numerical simulations. *Renew. Energy* **237**, 121671 (2024).

23. N. Christiansen, J. R. Carpenter, U. Daewel, N. Suzuki, C. Schrum, The large-scale impact of anthropogenic mixing by offshore wind turbine foundations in the shallow North Sea. *Front. Mar. Sci.* **10**, 1178330 (2023).

24. W. Shaw, L. Berg, M. Debnath, G. Deskos, C. Draxl, V. Ghate, C. Hasager, R. Kotamarthi, J. Mirocha, P. Muradyan, W. Pringle, D. Turner, J. Wilczak, Scientific challenges to characterizing the wind resource in the marine atmospheric boundary layer. *Wind Energy Sci.* **7**, 2307–2334 (2022).

25. P. Veers, K. Dykes, S. Basu, A. Bianchini, A. Clifton, P. Green, H. Holttinen, L. Kitzing, B. Kosovic, J. K. Lundquist, J. Meyers, M. O'Malley, W. J. Shaw, B. Straw, Grand challenges: Wind energy research needs for a global energy transition. *Wind Energy Sci.* **7**, 2491–2496 (2022).

26. R. M. Dorrell, C. J. Lloyd, B. J. Lincoln, T. P. Rippeth, J. R. Taylor, C.-c. P. Caulfield, J. Sharples, J. A. Polton, B. D. Scannell, D. M. Greaves, R. A. Hall, J. H. Simpson, Anthropogenic mixing in seasonally stratified shelf seas by offshore wind farm infrastructure. *Front. Mar. Sci.* **9**, 830927 (2022).

27. J. R. Carpenter, A. Guha, Blocking effects on mean ocean currents by offshore wind farm foundations. *Phys. Rev. Fluids* **9**, 103802 (2024).

28. B. Zhao, E. Sahlée, J. Du, L. Wu, Wind stress modification by offshore wind turbines: A numerical study of wave blocking impacts. *Ocean Eng.* **313**, 119651 (2024).

29. N. Bodini, J. K. Lundquist, A. Kirincich, U.S. East coast lidar measurements show offshore wind turbines will encounter very low atmospheric turbulence. *Geophys. Res. Lett.* **46**, 5582–5591 (2019).

30. N. Bodini, J. K. Lundquist, A. Kirincich, Offshore wind turbines will encounter very low atmospheric turbulence. *J. Phys. Conf. Ser.* **1452**, 012023 (2020).

31. B. Djath, J. Schulz-Stellenfleth, B. Cañadillas, Impact of atmospheric stability on X-band and C-band synthetic aperture radar imagery of offshore windpark wakes. *J. Renew. Sustain. Energy* **10**, 043301 (2018).

32. B. Cañadillas, R. Foreman, V. Barth, S. Siedersleben, A. Lampert, A. Platis, B. Djath, J. Schulz-Stellenfleth, J. Bange, S. Emeis, T. Neumann, Offshore wind farm wake recovery: Airborne measurements and its representation in engineering models. *Wind Energy* **23**, 1249–1265 (2020).

33. C. L. Archer, S. Wu, Y. Ma, P. A. Jiménez, Two corrections for turbulent kinetic energy generated by wind farms in the WRF model. *Mon. Weather Rev.* **148**, 4823–4835 (2020).

34. N. Bodini, J. K. Lundquist, P. Moriarty, Wind plants can impact long-term local atmospheric conditions. *Sci. Rep.* **11**, 22939 (2021).

35. R. Small, S. deSzoeke, S. Xie, L. O'Neill, H. Seo, Q. Song, P. Cornillon, M. Spall, S. Minobe, Air–sea interaction over ocean fronts and eddies. *Dynam. Atmos. Ocean* **45**, 274–319 (2008).

36. H. Seo, H. Song, L. W. O'Neill, M. R. Mazloff, B. D. Cornuelle, Impacts of ocean currents on the South Indian Ocean extratropical storm track through the relative wind effect. *J. Clim.* **34**, 9093–9113 (2021).

37. W. Sweet, R. Fett, J. Kerling, P. L. Violette, Air-sea interaction effects in the lower troposphere across the north wall of the gulf stream. *Mon. Weather Rev.* **109**, 1042–1052 (1981).

38. J. M. Wallace, T. P. Mitchell, C. Deser, The influence of sea-surface temperature on surface wind in the Eastern Equatorial Pacific: Seasonal and interannual variability. *J. Clim.* **2**, 1492–1499 (1989).

39. M. B. Christiansen, C. B. Hasager, Wake effects of large offshore wind farms identified from satellite SAR. *Remote Sens. Environ.* **98**, 251–268 (2005).

40. A. Platis, J. Bange, K. Bärfuss, B. Cañadillas, M. Hundhausen, B. Djath, A. Lampert, J. Schulz-Stellenfleth, S. Siedersleben, T. Neumann, S. Emeis, Long-range modifications of the wind field by offshore wind parks – Results of the project WIPAFF. *Meteorol. Z.* **29**, 355–376 (2020).

41. J. B. Olson, J. S. Kenyon, W. A. Angevine, J. M. Brown, M. Pagowski, K. Sušelj, *A Description of the MYNN-EDMF Scheme and the Coupling to Other Components in WRF–ARW* [Earth System Research Laboratory (U.S.), Global Systems Division, 2019], https://repository.library.noaa.gov/view/noaa/19837.

42. A. Abraham, M. Puccioni, A. Jordan, E. Maric, N. Bodini, N. Hamilton, S. Letizia, P. M. Klein, E. Smith, S. Wharton, J. Gero, J. D. Jacob, R. Krishnamurthy, R. K. Newsom, M. Pekour, P. Moriarty, Operational wind plants increase planetary boundary layer height: An observational study. *Wind Energy Sci.* **10**, 1681–1705 (2024).

43. J. M. Tomaszewski, J. K. Lundquist, Simulated wind farm wake sensitivity to configuration choices in the Weather Research and Forecasting model version 3.8.1. *Geosci. Model Dev.* **13**, 2645–2662 (2020).

44. S. Wu, C. L. Archer, Near-ground effects of wind turbines: Observations and physical mechanisms. *Mon. Weather Rev.* **149**, 879–898 (2021).

45. E. D. Christensen, M. Johnson, O. R. Sørensen, C. B. Hasager, M. Badger, S. E. Larsen, Transmission of wave energy through an offshore wind turbine farm. *Coast. Eng.* **82**, 25–46 (2013).

46. K. Bärfuss, J. Schulz-Stellenfleth, A. Lampert, The impact of offshore wind farms on sea state demonstrated by airborne LiDAR measurements. *J. Mar. Sci. Eng.* **9**, 644 (2021).

47. R. Castelao, S. Glenn, O. Schofield, Temperature, salinity, and density variability in the central Middle Atlantic Bight. *J. Geophys. Res. Oceans* **115**, C10005 (2010).

48. C. Cai, Y.-O. Kwon, Z. Chen, P. Fratantoni, Mixed layer depth climatology over the northeast U.S. continental shelf (1993–2018). *Cont. Shelf Res.* **231**, 104611 (2021).

49. E. Ludewig, "On the effect of offshore wind farms on the atmosphere and ocean dynamics" in *Hamburg Studies on Maritime Affairs* (Springer International Publishing, 2015), vol. 31.

50. R. K. Shearman, S. J. Lentz, Long-term sea surface temperature variability along the U.S. East Coast. *J. Phys. Oceanogr.* **40**, 1004–1017 (2010).

51. K. Chen, G. Gawarkiewicz, Y.-O. Kwon, W. G. Zhang, The role of atmospheric forcing versus ocean advection during the extreme warming of the Northeast U.S. continental shelf in 2012. *J. Geophys. Res. Oceans* **120**, 4324–4339 (2015).

52. H. Seo, L. W. O'Neill, M. A. Bourassa, A. Czaja, K. Drushka, J. B. Edson, B. Fox-Kemper, I. Frenger, S. T. Gille, B. P. Kirtman, S. Minobe, A. G. Pendergrass, L. Renault, M. J. Roberts, N. Schneider, R. J. Small, A. Stoffelen, Q. Wang, Ocean mesoscale and frontal-scale ocean–atmosphere interactions and influence on large-scale climate: A review. *J. Clim.* **36**, 1981–2013 (2023).

53. S. Redfern, M. Optis, G. Xia, C. Draxl, Offshore wind energy forecasting sensitivity to sea surface temperature input in the Mid-Atlantic. *Wind Energy Sci.* **8**, 1–23 (2023).

54. C. Wu, Q. Wang, K. Luo, J. Fan, Mesoscale impact of the sea surface on the performance of offshore wind farms. *J. Clean. Prod.* **372**, 133741 (2022).

55. L. F. Pareja-Roman, T. Miles, S. Glenn, Coastal upwelling modulates winds and air-sea fluxes, impacting offshore wind energy. *Front. Energy Res.* **12**, 1470712 (2024).

56. S. Baidya Roy, J. J. Traiteur, Impacts of wind farms on surface air temperatures. *Proc. Natl. Acad. Sci. U.S.A.* **107**, 17899–17904 (2010).

57. A. C. Fitch, J. K. Lundquist, J. B. Olson, Mesoscale influences of wind farms throughout a diurnal cycle. *Mon. Weather Rev.* **141**, 2173–2198 (2013).

58. D. A. Rajewski, E. S. Takle, J. K. Lundquist, S. Oncley, J. H. Prueger, T. W. Horst, M. E. Rhodes, R. Pfeiffer, J. L. Hatfield, K. K. Spoth, R. K. Doorenbos, Crop wind energy experiment (CWEX): Observations of surface-layer, boundary layer, and mesoscale interactions with a wind farm. *Bull. Am. Meteorol. Soc.* **94**, 655–672 (2013).

59. L. Zhou, Y. Tian, S. Baidya Roy, C. Thorncroft, L. F. Bosart, Y. Hu, Impacts of wind farms on land surface temperature. *Nat. Clim. Change* **2**, 539–543 (2012).

60. Weather, "Climatic and ecological impacts of onshore wind farms" in *Comprehensive Renewable Energy* (Elsevier, 2022), pp. 165–188.

61. G. Xia, L. Zhou, J. M. Freedman, S. B. Roy, R. A. Harris, M. C. Cervarich, A case study of effects of atmospheric boundary layer turbulence, wind speed, and stability on wind farm induced temperature changes using observations from a field campaign. *Clim. Dynam.* **46**, 2179–2196 (2016).

62. B. Thomas, X. Costoya, M. deCastro, D. Carvalho, M. Gómez-Gesteira, Wake effect impact on the levelized cost of energy in large floating offshore wind farms: A case of study in the northwest of the Iberian Peninsula. *Energy* **304**, 132159 (2024).

63. T. W. Juliano, B. Kosović, P. A. Jiménez, M. Eghdami, S. E. Haupt, A. Martilli, "Gray zone" simulations using a three-dimensional planetary boundary layer parameterization in the weather research and forecasting model. *Mon. Weather Rev.* **150**, 1585–1619 (2022).

64. W. G. Large, J. C. McWilliams, S. C. Doney, Oceanic vertical mixing: A review and a model with a nonlocal boundary layer parameterization. *Rev. Geophys.* **32**, 363–403 (1994).

65. L. Johnson, B. Fox-Kemper, Q. Li, H. T. Pham, S. Sarkar, A finite-time ensemble method for mixed layer model comparison. *J. Phys. Oceanogr.* **53**, 2211–2230 (2023).

66. W. C. Skamarock, J. B. Klemp, J. Dudhia, D. O. Gill, Z. Liu, J. Berner, W. Wang, J. G. Powers, M. G. Duda, D. M. Barker, X.-Y. Huang, A Description of the Advanced Research WRF Model Version 4.3 (no. NCAR/TN-556+STR) (2021), doi:10.5065/1dfh-6p97.

67. A. F. Shchepetkin, J. C. McWilliams, The regional oceanic modeling system (ROMS): A split-explicit, free-surface, topography-following-coordinate oceanic model. *Ocean Model.* **9**, 347–404 (2005).

68. H. L. Tolman, B. Balasubramaniyan, L. D. Burroughs, D. V. Chalikov, Y. Y. Chao, H. S. Chen, V. M. Gerald, Development and implementation of wind-generated ocean surface wave modelsat NCEP. *Weather and Forecasting* **17**, 311–333 (2002).

69. H. Seo, A. J. Miller, J. O. Roads, The Scripps Coupled Ocean–Atmosphere Regional (SCOAR) model, with applications in the Eastern Pacific sector. *J. Clim.* **20**, 381–402 (2007).

70. C. Sauvage, H. Seo, C. A. Clayson, J. B. Edson, Improving wave-based air-sea momentum flux parameterization in mixed seas. *J. Geophys. Res. Oceans* **128**, e2022JC019277 (2023).

71. C. W. Fairall, E. F. Bradley, J. E. Hare, A. A. Grachev, J. B. Edson, Bulk parameterization of air–sea fluxes: Updates and verification for the COARE algorithm. *J. Clim.* **16**, 571–591 (2003).

72. J. B. Edson, V. Jampana, R. A. Weller, S. P. Bigorre, A. J. Plueddemann, C. W. Fairall, S. D. Miller, L. Mahrt, D. Vickers, H. Hersbach, On the exchange of momentum over the open ocean. *J. Phys. Oceanogr.* **43**, 1589–1610 (2013).

73. J. B. Olson, *A Description of the MYNN Surface-Layer Scheme* [Global Systems Laboratory (U.S.), 2021], https://repository.library.noaa.gov/view/noaa/30605.

74. C. Sauvage, H. Seo, B. W. Barr, J. B. Edson, C. A. Clayson, Misaligned wind-waves behind atmospheric cold fronts. *J. Geophys. Res. Oceans* **129**, e2024JC021162 (2024).

75. H. Hersbach, B. Bell, P. Berrisford, S. Hirahara, A. Horányi, J. Muñoz Sabater, J. Nicolas, C. Peubey, R. Radu, D. Schepers, A. Simmons, C. Soci, S. Abdalla, X. Abellan, G. Balsamo, P. Bechtold, G. Biavati, J. Bidlot, M. Bonavita, G. De Chiara, P. Dahlgren, D. Dee, M. Diamantakis, R. Dragani, J. Flemming, R. Forbes, M. Fuentes, A. Geer, L. Haimberger, S. Healy, R. J. Hogan, E. Hólm, M. Janisková, S. Keeley, P. Laloyaux, P. Lopez, C. Lupu, G. Radnoti, P. De Rosnay, I. Rozum, F. Vamborg, S. Villaume, J.-N. Thépaut, The ERA5 global reanalysis. *Q. J. R. Meteorol. Soc.* **146**, 1999–2049 (2020).

76. P. Weatherall, K. M. Marks, M. Jakobsson, T. Schmitt, S. Tani, J. E. Arndt, M. Rovere, D. Chayes, V. Ferrini, R. Wigley, A new digital bathymetric model of the world's oceans. *Earth Space Sci.* **2**, 331–345 (2015).

77. J.-M. Lellouche, E. Greiner, O. Le Galloudec, G. Garric, C. Regnier, M. Drevillon, M. Benkiran, C.-E. Testut, R. Bourdalle-Badie, F. Gasparin, O. Hernandez, B. Levier, Y. Drillet, E. Remy, P.-Y. Le Traon, Recent updates to the Copernicus Marine Service global ocean monitoring and forecasting real-time 1/12° high-resolution system. *Ocean Sci.* **14**, 1093–1126 (2018).

78. N. Rascle, F. Ardhuin, A global wave parameter database for geophysical applications. Part 2: Model validation with improved source term parameterization. *Ocean Model.* **70**, 174–188 (2013).

79. G. D. Egbert, S. Y. Erofeeva, Efficient inverse modeling of barotropic ocean tides. *J. Atmos. Ocean. Technol.* **19**, 183–204 (2002).

80. J. Steffen, H. Seo, C. A. Clayson, S. Pei, T. Shinoda, Impacts of tidal mixing on diurnal and intraseasonal air-sea interactions in the Maritime Continent. *Deep Sea Res. 2 Top. Stud. Oceanogr.* **212**, 105343 (2023).

81. J. K. Lundquist, K. K. DuVivier, D. Kaffine, J. M. Tomaszewski, Costs and consequences of wind turbine wake effects arising from uncoordinated wind energy development. *Nat. Energy* **4**, 26–34 (2018).

82. C. S. Bretherton, M. Widmann, V. P. Dymnikov, J. M. Wallace, I. Bladé, The effective number of spatial degrees of freedom of a time-varying field. *J. Clim.* **12**, 1990–2009 (1999).

83. A. Monin, A. Obukhov, Basic laws of turbulent mixing in the surface layer of the atmosphere (in Russian) (Tr. Akad. Nauk SSSR Geophiz. Inst.), vol. 24 (151), pp. 163–187 (1954), https://gibbs.science/efd/handouts/monin_obukhov_1954.pdf.

84. D. Quint, J. K. Lundquist, D. Rosencrans, Simulations suggest offshore wind farms modify low-level jets. *Wind Energy Sci.* **10**, 117–142 (2025).

85. S.-B. Kim, I. Fukumori, T. Lee, The closure of the ocean mixed layer temperature budget using level-coordinate model fields. *J. Atmos. Ocean. Technol.* **23**, 840–853 (2006).

86. R. W. Reynolds, N. A. Rayner, T. M. Smith, D. C. Stokes, W. Wang, An improved in situ and satellite SST analysis for climate. *J. Clim.* **15**, 1609–1625 (2002).

87. J. S. Kain, The Kain–Fritsch convective parameterization: An update. *J. Appl. Meteorol. Climatol.* **43**, 170–181 (2004).

88. G. Thompson, P. R. Field, R. M. Rasmussen, W. D. Hall, Explicit forecasts of winter precipitation using an improved bulk microphysics scheme. Part II: Implementation of a new snow parameterization. *Mon. Weather Rev.* **136**, 5095–5115 (2008).

89. M. Tewari, F. Chen, W. Wang,J. Dudhia, M. LeMone, K. Mitchell, M. Ek, G. Gayno, J. Wegiel, R. Cuenca, Implementation and verification of the unified NOAH land surface model in the WRF model, In *Proceedings of the 20th Conference on Weather Analysis and Forecasting/16th Conference on Numerical Weather Prediction*, Seattle, WA, USA, 2004, vol. 14, pp. 11–15.

90. M. J. Iacono, J. S. Delamere, E. J. Mlawer, M. W. Shephard, S. A. Clough, W. D. Collins, Radiative forcing by long-lived greenhouse gases: Calculations with the AER radiative transfer models. *J. Geophys. Res. Atmos.* **113**, D13103 (2008).

91. M. Nakanishi, H. Niino, Development of an improved turbulence closure model for the atmospheric boundary layer. *J. Meteorol. Soc. Japan. Ser. II* **87**, 895–912 (2009).

92. J. C. Warner, C. R. Sherwood, H. G. Arango, R. P. Signell, Performance of four turbulence closure models implemented using a generic length scale method. *Ocean Model.* **8**, 81–113 (2005).

93. V. M. Canuto, A. Howard, Y. Cheng, M. S. Dubovikov, Ocean turbulence. Part I: One-point closure model—Momentum and heat vertical diffusivities. *J. Phys. Oceanogr.* **31**, 1413–1426 (2001).

94. F. Ardhuin, B. Chapron, F. Collard, Observation of swell dissipation across oceans. *Geophys. Res. Lett.* **36**, 2008GL037030 (2009).

95. F. Ardhuin, E. Rogers, A. V. Babanin, J.-F. Filipot, R. Magne, A. Roland, A. Van Der Westhuysen, P. Queffeulou, J.-M. Lefevre, L. Aouf, F. Collard, Semiempirical dissipation source functions for ocean waves. Part I: Definition, calibration, and validation. *J. Phys. Oceanogr.* **40**, 1917–1941 (2010).

96. F. Ardhuin, A. Roland, Coastal wave reflection, directional spread, and seismoacoustic noise sources. *J. Geophys. Res.* **117**, C00J20 (2012).

97. J. A. Battjes, J. P. F. M. Janssen, "Energy loss and set-up due to breaking of random waves" in *Coastal Engineering 1978* (American Society of Civil Engineers, 1978), pp. 569–587, http://ascelibrary.org/doi/10.1061/9780872621909.034.

98. F. Ardhuin, W. C. O'Reilly, T. H. C. Herbers, P. F. Jessen, Swell transformation across the continental shelf. Part I: Attenuation and directional broadening. *J. Phys. Oceanogr.* **33**, 1921–1939 (2003).
